# Changes in life satisfaction, self-esteem, and self-rated health before, during, and after becoming a young carer in the UK: a longitudinal, propensity score analysis

**DOI:** 10.1016/j.lanepe.2024.101187

**Published:** 2024-12-20

**Authors:** Rebecca E. Lacey, Alejandra Letelier, Baowen Xue, Anne McMunn

**Affiliations:** a School of Health and Medical Sciences, City St George's, University of London, Cranmer Terrace, London, SW17 0RE, UK; b Research Department of Epidemiology and Public Health, University College London, 1-19 Torrington Place, London, WC1E 6BT, UK

**Keywords:** Young carer, Unpaid caring, Wellbeing, Longitudinal, Life course, Transitions, Life satisfaction, Self-esteem, Self-rated health

## Abstract

**Background:**

The health of young carers is poorer, on average, than their peers. The timing and persistence of health and wellbeing changes around becoming a young carer are unknown. We investigated how health and wellbeing change before, during and after becoming a young carer in the UK and whether this varies by caring intensity, age, gender, ethnicity, or household income.

**Methods:**

We used data from the UK Household Longitudinal Study (2009–2023) on young people aged 10–25. Outcomes were self-rated health, life satisfaction and self-esteem (8-item Rosenberg scale). We used propensity score matching to match young carers to similar non-carers and applied piecewise growth curve modelling to model health and wellbeing trajectories for young carers and non-carers. Analyses were stratified by caring intensity (hours and recipient), age, gender, household income and ethnicity. Samples varied from 2320 (self-esteem by age-group) to 4606 (self-rated health by household income).

**Findings:**

Approximately 12% (n = 2400/16,622) of young people became young carers. Young carers had lower life satisfaction two years prior to becoming a young carer (−0.03, 95% confidence interval: −0.09, −0.01) and this difference persisted for three years after. Young carers who cared for 10 or more hours/week (−0.03, 95% confidence interval: −0.10, 0.04), those from Black ethnic groups (−0.22, 95% confidence interval: −0.38, −0.05), and those from households in the lowest fifth of income had larger differences in life satisfaction before and during becoming a young carer (−0.05, 95% confidence interval: −0.13, 0.04). We observed no differences in self-esteem or self-rated health during or after becoming a young carer.

**Interpretation:**

These findings highlight the importance of early identification and support for young carers plus reducing the care loads of young carers to prevent declines in wellbeing.

**Funding:**

The project has been funded by the 10.13039/501100000279Nuffield Foundation and the Joint Programming Initiative More Years Better Lives from the national funding body UK 10.13039/501100000269Economic and Social Research Council.

**Research in context**

**Evidence before this study**

PubMed was searched on 23rd May 2024 to identify longitudinal studies examining health and wellbeing changes around becoming a young carer. Titles and abstracts were searched using the following terms: ((caregiv∗) OR (carer∗)) AND ((health) OR (wellbeing)) AND ((longitudinal) OR (cohort)) AND ((transition) OR (uptak∗) OR (becom∗)). Eligible articles were those published in English, from any year and if they quantified the health or wellbeing effects of becoming a young carer. No articles were identified. All prior studies have examined changes in health and wellbeing in carers of other ages, particularly in age 50+.

**Added value of this study**

This study is the only one to date to examine how health and wellbeing change around becoming a young carer. In addition, we assessed how any changes observed vary by caregiving intensity, age, gender, ethnicity, and household income. To do this we used high-quality UK household panel data on individuals aged 10–25. Our results suggest that life satisfaction (but not self-esteem or self-rated health) declines modestly but quickly after becoming a young carer and differences in life satisfaction begin to emerge two years prior to becoming a young carer and persist for three years after becoming a young carer. This was particularly the case for young carers who cared for more than 10 h per week, were from Black ethnic groups, or were from lower income households. Results did not differ by age group or gender.

**Implications of all the available evidence**

Our findings provide evidence for the importance of early identification and support for young carers. This support should have a particular focus on reducing the amount of care a young carer is providing and supporting their wellbeing to prevent long-term impacts. Health professionals, social work practitioners and staff in education institutions would be well situated in young carer identification efforts. Governments could mandate the requirement for health professionals supporting adults with long-term health and support needs to ask whether there are any young people who are providing care and make appropriate referrals for support.

## Introduction

An unpaid or informal carer is someone who provides support to a family or friend due to illness (mental or physical), disability or addiction, who cannot cope without this help.^1^ Informal, unpaid caring constitutes a significant proportion of social care provision internationally. For instance, in Europe, 80% of long-term care is provided by unpaid or informal carers.^1^^,^^2^ Young carers (aged <18) are an important, but often hidden, part of unpaid caring in many countries and subsequently, countries vary greatly in their recognition and support for young carers.^3^ Around 12% of young people in the United Kingdom (UK) report having care responsibilities—an increase from ∼8% before the Covid-19 pandemic.^4^ Young carers most commonly report caring for parents, grandparents and siblings,^4^^,^^5^ and navigate caring alongside schoolwork, friendships, puberty, and increasing challenges to young peoples’ mental health.^6^

Two systematic reviews^7^^,^^8^ showed that the health of young carers is poorer, on average, than their peers, but very little longitudinal research on young carers’ health exists. No study has examined how health and wellbeing change around becoming a young carer—a question reliant on longitudinal data. Studies which have assessed health and wellbeing around becoming a young adult carer (aged 16–29) in the UK showed that mental (but not physical) health and wellbeing changes around becoming a carer,^9^^,^^10^ with effects marked for carers who provided more care (>10 h/week).^9^^,^^10^ In fact, two previous studies using the UK Household Longitudinal Study (UKHLS) found that life satisfaction and health functioning declined before reporting becoming a carer, consistent with the “caregiver career” where an individual is providing care but does not yet recognise themselves as a carer.^11^ All other prior studies looking at health and wellbeing changes focused on older carers only, finding that health^12^ and wellbeing^13^, ^14^, ^15^ worsened for carers. It cannot be assumed that the health and wellbeing effects of becoming a young carer are the same as at older ages, particularly when young caring occurs during a life stage where caring is less normative and when caring for a parent (the most reported care recipient for young carers) results in parentification.^3^

There are important social inequalities in young caring,^4^^,^^5^ yet we do not know how inequalities by gender, ethnicity, and household income impact upon health and wellbeing around becoming a young carer. One previous longitudinal study from Australia showed that the association between young caring and mental health did not differ by gender,^16^ but we do not know whether this extends to broader measures of health and wellbeing, nor to change around becoming a young carer. No prior studies have looked at inequalities in young carers' health by ethnicity or household income.^7^ We might anticipate that changes in health and wellbeing around becoming a young carer might be more pronounced in minoritised ethnic groups and those with lower household income. Finally, it is important to look at age differences. Policy and practice organisations consider young carers to be ≤25 years as the support needs for those aged 18–25 are closer to young carers (<18 years) than older carers. However, in many countries’ policies young carers are typically considered to be <18 years and young adult carers aged 18–25 years. It is important to examine differences by age, as the context for those <18 years, who are typically in school, is different from those aged 18+.

The aim of this study was to examine how health and wellbeing (life satisfaction, self-esteem and self-rated health) change before, during and after becoming a young carer and whether this varied by caregiving intensity (hours spent caring per week and care recipient), age group, gender, ethnicity, and household income. We hypothesised that: 1) health and wellbeing trajectories would begin to worsen shortly before becoming a young carer compared to matched non-carers; 2) changes in health and wellbeing would be more pronounced for young people who were younger, caring for more hours, caring for a parent, female, from an ethnic minority group, or with lower levels of household income.

## Methods

### Data

This study used data from the UK Household Longitudinal Study (UKHLS), or “Understanding Society”. The UKHLS, a UK-representative household panel, began in 2009 with approximately 40,000 households utilising a stratified, clustered probability sampling design. All household members aged 10+ are interviewed annually. Those aged 10–15 via a youth questionnaire and people aged 16+ via an adult questionnaire. This study used data from waves 1 to 13 (2009–2023) and focuses on young people aged 10–25. Response rates are acceptable over time; at wave 13 the youth questionnaire response rate was 56% and the adult questionnaire 65%.^17^ However, the general population sample of UKHLS lost 60.1% of its wave 1 sample between waves 2–11.^18^ Survey participants provided informed consent and the UKHLS has ethical approval from the Ethics Committee of the University of Essex. UKHLS data are available via the UK Data Service.

The study protocol was pre-registered: https://doi.org/10.17605/OSF.IO/VEZXN.

### Measures

Young people aged 10–15 were asked “Some people your age may have to look after other people. This could be a brother or sister, a relative or someone else who is disabled or sick. Is there anyone like this who lives here with you that you have to look after on a regular basis?” This information was collected every alternate wave from waves 3 to 13. Due to concerns about potentially including babysitting, we only included young people who reported solely caring for a child if an adult in the household was also a carer. Young carers reporting care for any other relation or to a child plus someone else were considered young carers. Those aged 16–25 were asked “Is there anyone living with you who is sick, disabled or elderly whom you look after or give special help to (for example, a sick, disabled or elderly relative, husband, wife or friend etc)?” This information was collected annually. Young people who answered yes to either question were considered as young carers. Young carers were also asked how many hours of care per week they provided which was categorised as <10 h and 10+ hours per week based on prior work. Young carers were asked who they provided care to and we categorised this as parent vs other.

Two measures of wellbeing were included—life satisfaction and self-esteem. Life satisfaction was assessed in all waves. In the adult questionnaire the question was “Please choose the number which you feel best describes how dissatisfied or satisfied you are with the following aspects of your current situation. Your life overall” Responses were collected via a seven-point Likert scale from Completely dissatisfied (1) to Completely satisfied (7). In the youth questionnaire the question was “Which best describes how you feel about your life as a whole?” and respondents were asked to select the emoticon which best reflected how they felt from very happy (1) to very unhappy (7). This scale was reverse coded so that life satisfaction was scored from low to high.

Regarding self-esteem, the UKHLS included the eight-item Rosenberg self–esteem scale^19^ in alternate waves from wave 2 in the adult and youth questionnaires for participants aged 10–21. Example items included “I am a likeable person” and “I feel I have a number of good qualities”. The internal consistency across waves ranged from 0.74 to 0.86. Responses to each item ranged from Strongly disagree (1) to Strongly agree (4). Responses were summed to create a score ranging from 4 to 32 with higher scores indicating higher self-esteem.

Finally, participants were asked “In general, would you say your health is Excellent (1), Very Good (2), Good (3), Fair (4), and Poor (5)?” In the adult questionnaire this was asked every year and every alternate year from wave 2 in the youth questionnaire. This variable was dichotomised into “Poor health” (Fair/Poor, 0) and “Good health” (Excellent/Very Good/Good, 1), consistent with the direction of the wellbeing scores. The Appendix (p.2) shows the availability of young caring and outcome measurements across waves.

Covariates were taken from the first wave of observation and included age, gender, ethnicity, household income, number of siblings in the household, family structure, parental occupational social class, parental education, urbanicity, and number of waves participated in (Appendix, p.3). We use the term gender because we are studying care which has traditionally been ascribed to women more than men.

### Statistical analysis

We used propensity score matching (PSM) to reduce pre-care differences between young people who became young carers and those who did not. We matched each young carer to two similar non-carers based on the covariates above using nearest neighbour matching without replacement, and with exact matching on gender. For the inequalities analysis we removed the covariate from the PSM which was our focus in that analysis, for instance for analyses looking at inequalities by ethnicity we did not include ethnicity in the PSM but did include it when examining gender, age group, caring intensity and income inequalities. To assess the PSM performance we checked the balance in distribution across all analysis variables before and after PSM, and created propensity score density plots for carers and non-carers before and after matching (Appendix p.4).

Health and wellbeing trajectories before, during and after becoming a young carer: Piecewise linear growth curves were used to estimate the average trajectories of health and wellbeing before, during and after becoming a young carer with trajectories centred on the point of becoming a young carers (non-carers assigned the same caring transition point as their matched young carers). This allowed us to estimate trajectories of health and wellbeing up to 12 years before and after becoming a young carer. Due to limited data at the most distant timepoints, these years were excluded from our analyses. The models included life satisfaction trajectories from −11 to +10 years, self-rated health trajectories from −10 to +9 years, and self-esteem trajectories from −8 to +8 years from the transition to young caring. Wellbeing trajectories were predicted using average marginal effects at each year (with 95% CIs) and average predicted probabilities for reporting very good/good health (with 95% CIs) at each year.

To formally test for changes in health and wellbeing when becoming a young carer and differences in the subsequent slopes we partitioned the trajectories into three segments (−11 to −1 years, pre-caring; −1 to 0 years, transition to caring; 1–11 years, post-transition) with turning points set at years −1 and 0. Using multilevel mixed-effects linear regression we tested interaction terms to observe whether changes differed between young carers and non-carers. Extending this method, we tested differences by gender, ethnicity, age group and household income using three-way interaction terms. Differences by care intensity were tested only among young carers, with the interaction term being between caring hours/recipient and slope change. We plotted the marginal effects of time on wellbeing to illustrate the changes in health and wellbeing before, during and after the onset of young caring for each group (Appendix p. 9).

All analyses were conducted using Stata v.18.

The study includes all young people who provided information on caring in at least one wave between ages 10 and 25 years. The analyses were focused on participants’ first transition into young caring during this range. We excluded participants with missing information on baseline covariates which were essential for the PSM and those who did not have at least one measure of health or wellbeing before and after young carer transition. The sample selection process is detailed in the Appendix (p. 10). The final sample sizes range from 2320 (self-esteem stratified by age group) to 4606 (self-rated health stratified by household income).

The funder played no role in the design, analysis, interpretation of results, or the decision to submit this work for publication.

## Results

The baseline characteristics of study participants are shown in the Appendix (pp. 5–7, pre-PSM columns). Depending on the specific sample being used, between 11.2 and 12.6% of 10–25-year-olds became young carers. Most young carers reported caring for <10 h per week, and most were caring for a parent. Young carers were equally distributed by gender but more 10–17-year-olds as opposed to 18–25-year-olds became young carers. Compared to non-carers, young carers were more likely to come from Pakistani and Bangladeshi, and Black ethnic minorities. Young carers were also more likely to live in urban areas, be from single parent households, have more siblings, come from lower income households, have parents who were not working, and have parents with lower or no educational qualifications.

### Becoming a young carer and life satisfaction

**Fig. 1:**
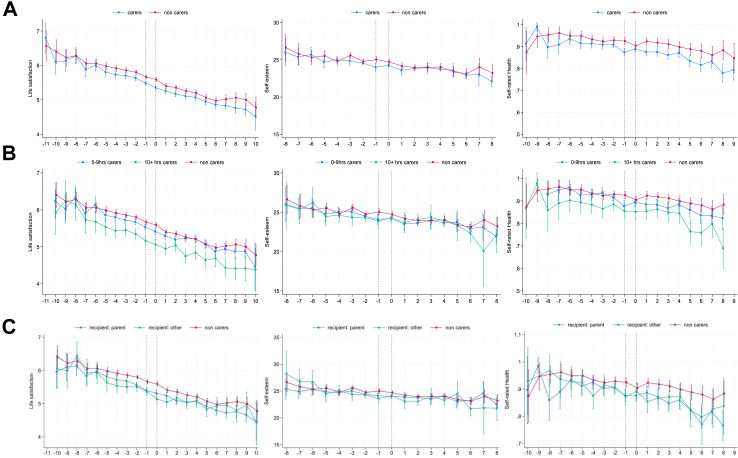
**Changes in life satisfaction, self-esteem, and self-rated health before, during and after becoming a young carer (aged 10–25) in the UK and stratified by caring hours and recipient**. A Trajectories of life satisfaction, self-esteem and self-rated health before, during and after becoming a young carer. B Trajectories of life satisfaction, self-esteem and self-rated health before, during and after becoming a young carer stratified by caring hours per week. C Trajectories of life satisfaction, self-esteem and self-rated health before, during and after becoming a young carer stratified by care recipient (parent vs other) x-axis is years. Dotted vertical lines indicate the transition to young caring from −1 to 0 years. Higher y-axis values represent better predicted mean life satisfaction and self-esteem, and higher probability of reporting good/very good health. Self-esteem analyses only pertain to 10–21 year olds due to availability of this measure in this age group only.

Fig. 1 (panel A) shows that young carers reported a small but significant decrease in life satisfaction upon becoming a young carer relative to non-carers (−0.03, 95% CI: −0.09, −0.01; Table 1). The decline in life satisfaction was equivalent to a −0.13-point difference during the transition year (labelled -1-0) (Appendix p. 13). In addition, the difference in life satisfaction between young carers and their peers appears two years prior to becoming a young carer. Post transition, the change in life satisfaction was different between young carers and their peers (0.05, 95% CI: −0.01, 0.10). Differences in life satisfaction that emerged shortly before becoming a young carer persisted for three years after becoming a young carer. Stratifying by caring intensity (Fig. 1, panels B and C; Table 1), the decline in life satisfaction observed for young carers during the transition did not vary by caring hours or care recipient, nor did post-transition slopes. However, there was a clear distinction in life satisfaction levels for young people who transitioned into caring for more than 10 h per week four years’ prior to becoming a young carer and lower levels of life satisfaction for this group persisted for five years after.

**Table 1:** Results of interaction analyses for life satisfaction.

	Care# transition slope	95% CI		p-value	Care# post transition slope	95% CI		p-value
**Care (non-carer ref)**								
Carer	−0.03	−0.09	−0.01	0.020	0.05	−0.01	0.10	0.054
**Caring hours (ref 1**–**9 h/week)**^a^								
10 or + hrs/week	−0.03	−0.10	0.04	0.332	0.03	−0.05	0.11	0.450
**Care recipient (ref parent)**^a^								
Other	0.02	−0.05	0.09	0.597	0.01	−0.08	0.08	0.994
**Age group (non-carer ref)**								
Carers 10–17	−0.03	−0.09	0.02	0.252	0.06	−0.01	0.12	0.078
Carers 18–25	−0.06	−0.15	0.02	0.090	0.09	−0.02	0.16	0.433
**Gender (non-carer ref)**								
Male carers	−0.03	−0.09	0.03	0.330	0.04	−0.03	0.10	0.305
Female carers	−0.03	−0.09	0.02	0.299	0.04	−0.03	0.11	0.228
**Ethnicity (non-carer ref)**								
White carers	−0.03	−0.07	0.03	0.272	0.04	−0.02	0.09	0.175
Black carers	−0.22	−0.38	−0.05	0.013	0.27	0.07	0.47	0.009
Indian carers	−0.03	−0.23	0.17	0.773	−0.01	−0.24	0.22	0.937
Pakistani/Bangladeshi carers	0.08	−0.06	0.21	0.282	−0.06	−0.22	0.10	0.476
Other Ethnic grp carers	0.19	−0.37	0.01	0.052	0.19	−0.04	0.41	0.098
**Hhold income (non-carer ref)**								
Highest income quintile carers	0.01	−0.10	0.11	0.909	−0.01	−0.13	0.12	0.950
Second quintile carers	0.01	−0.09	0.11	0.809	−0.01	−0.11	0.11	0.809
Third quintile carers	0.01	−0.08	0.09	0.903	0.02	−0.09	0.12	0.738
Fourth quintile carers	−0.01	−0.09	0.09	0.987	0.03	−0.07	0.13	0.581
Lowest quintile carers	−0.05	−0.13	0.04	0.298	0.07	−0.03	0.17	0.206

a Interaction only tested among young carers.

**Fig. 2:**
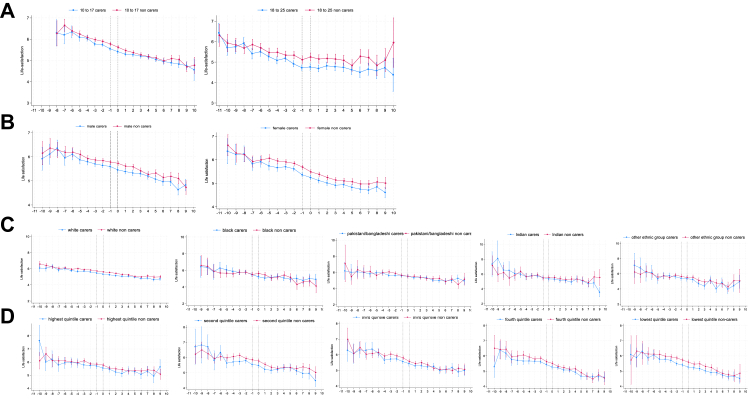
**Changes in life satisfaction before, during and after becoming a young carer (aged 10–25) stratified by gender, age group, ethnicity and household income**. A Trajectories of life satisfaction before, during and after becoming a young carer by gender. B Trajectories of life satisfaction before, during and after becoming a young carer by age group. C Trajectories of life satisfaction before, during and after becoming a young carer by ethnicity. D Trajectories of life satisfaction before, during and after becoming a young carer by household income. x-axis is years. Dotted vertical lines indicate the transition to young caring from −1 to 0 years. Higher values represent higher (more positive) predicted mean life satisfaction.

Regarding inequalities in life satisfaction (Fig. 2; Table 1), the changes in life satisfaction for young carers did not differ by age group (panel A) or gender (panel B). However, we found that amongst Black young people (Fig. 2, panel C; Table 1), young carers had a significant decline in life satisfaction when becoming a young carer (−0.22, 95% CI: −0.38, −0.05) which was stronger than that observed amongst White young carers (−0.03, 95% CI: −0.07, 0.03). The subsequent trajectory of life satisfaction, however, was more positive compared to non-carers (0.27, 95% CI: 0.07, 0.47). Among the Other ethnic group, there was a positive/flatter slope in life satisfaction during the transition to young caring relative to non-carers (0.19, 95% CI: −0.37, 0.01). No differences between young carers and their peers in life satisfaction trajectories were observed within other ethnic groups. For household income (Fig. 2, panel D) we observed no statistical differences in life satisfaction slopes during young carer transition or after (Table 1; Appendix p. 19). However, young carers from the lowest fifth of household income had lower levels of life satisfaction two years prior to becoming a young carer which persisted until two years after transition.

### Becoming a young carer and self-esteem

**Fig. 3:**
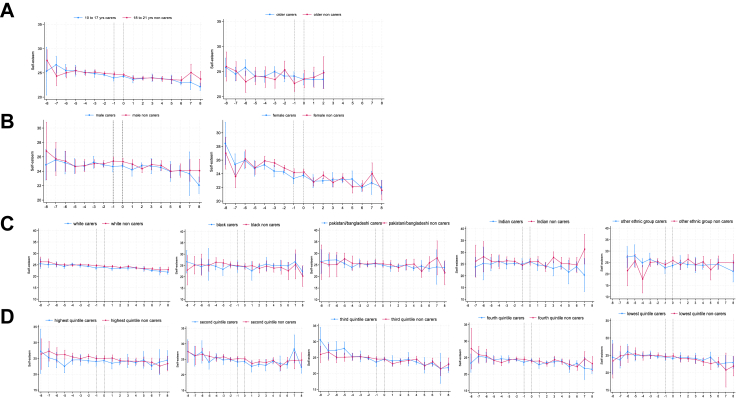
**Changes in self-esteem before, during and after becoming a young carer (aged 10–21) stratified by gender, age group, ethnicity and household income**. A Trajectories of self-esteem before, during and after becoming a young carer by gender. B Trajectories of self-esteem before, during and after becoming a young carer by age group. C Trajectories of self-esteem before, during and after becoming a young carer by ethnicity. D Trajectories of self-esteem before, during and after becoming a young carer by household income. x-axis is years. Dotted vertical lines indicate the transition to young caring from −1 to 0 years. Higher values represent higher predicted mean self-esteem.

Fig. 1 (panel A) shows self-esteem did not change during the transition to young caring compared to non-carers nor did it differ after young caring transition (Table 2; Appendix p. 14 and p. 19). However, self-esteem was lower, on average, at the timepoint before becoming a young carer. Trajectories of self-esteem did not vary by caring intensity (Fig. 1, panel B and C), nor by age, gender, ethnicity, or household income (Fig. 3).

**Table 2:** Results of interaction analyses for self-esteem.

	Care# transition slope	95% CI		p-value	Care# post transition slope	95% CI		p-value
**Care (non-carer ref)**								
Carer	0.12	−0.08	0.33	0.228	−0.13	−0.43	0.16	0.374
**Caring hours (ref 1**–**9 h/w)**^a^								
10 or + hrs/week	0.38	−0.01	0.75	0.054	−0.53	−1.07	0.02	0.058
**Care recipient (ref parent)**^a^								
Other	−0.17	−0.55	0.21	0.371	0.09	−0.46	0.65	0.742
**Age group (non-carer ref)**								
Carers 10–17	0.19	−0.04	0.41	0.100	−0.29	−0.62	0.04	0.087
Carers 18–25	−0.34	−1.37	0.69	0.517	−0.22	−4.10	3.66	0.912
**Gender (non-carer ref)**								
Male carers	0.08	−0.21	0.38	0.578	−0.09	−0.52	0.33	0.658
Female carers	0.23	−0.05	0.51	0.107	−0.24	−0.64	0.17	0.249
**Ethnicity (non-carer ref)**								
White carers	0.04	−0.19	0.27	0.721	−0.01	−0.34	0.32	0.943
Black carers	0.25	−0.59	1.09	0.561	0.01	−1.21	1.22	0.993
Indian carers	0.09	−0.94	1.12	0.864	−0.56	−2.09	0.95	0.468
Pakistani/Bangladeshi carers	−0.14	−0.63	0.91	0.718	−0.21	−1.34	0.91	0.709
Other Ethnic grp carers	−0.74	−1.79	0.29	0.158	1.08	−0.45	2.62	0.168
**Hhold income (non-carer ref)**								
Highest income quintile carers	0.41	−0.53	1.34	0.398	−0.29	−1.37	0.80	0.603
Second quintile carers	−0.24	−1.01	0.52	0.527	0.28	−0.60	1.17	0.529
Third quintile carers	0.39	−0.37	1.14	0.315	−0.48	−1.35	0.40	0.286
Fourth quintile carers	0.14	−0.53	0.80	0.690	−0.13	−0.91	0.64	0.739
Lowest quintile carers	−0.06	−0.79	0.67	0.875	0.18	−0.68	1.04	0.685

a Interaction only tested among young carers.

### Becoming a young carer and self-rated health

**Fig. 4:**
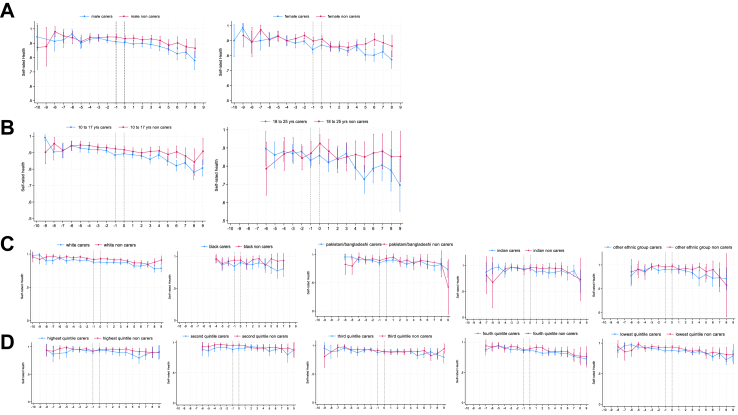
**Changes in self-rated health before, during and after becoming a young carer (aged 10–25) stratified by gender, age group, ethnicity and household income**. A Trajectories of self-rated health before, during and after becoming a young carer by gender. B Trajectories of self-rated health before, during and after becoming a young carer by age group. C Trajectories of self-rated health before, during and after becoming a young carer by ethnicity. D Trajectories of self-rated health before, during and after becoming a young carer by household income. x-axis is years. Dotted vertical lines indicate the transition to young caring from −1 to 0 years. Higher values represent higher predicted mean probabilities of reporting good/very good health.

Fig. 1 (panel A) shows self-rated health did not vary around or after the transition to young caring (Table 3). However, the predicted probability of reporting good health was lower at the timepoint immediately prior to becoming a young carer (Fig. 1, panel A; Appendix p. 15 and p. 20). There was no variation by caring hours or care recipient (Table 3; Fig. 1, panel B and C); however, young carers who started caring for 10+ hours per week had lower levels of health two years prior to becoming a young carer compared to young carers providing 0–9 h per week (Fig. 1, panel B). There were no differences by age, ethnicity, or household income (Fig. 4, panels A, C and D; Table 3). Regarding gender, the slopes in predicted health went in opposite directions for male and female young carers (male: −0.19, 95% CI: −0.63, 0.24; female: 0.32, 95% CI: −0.04, 0.67) and female young carers had lower levels of health at the two timepoints prior to reporting being a young carer (Fig. 4, panel B).

**Table 3:** Results of interaction analyses for self-rated health.

	Care# transition slope	95% CI		p-value	Care# post transition slope	95% CI		p-value
**Care (non-carer ref)**								
Carer	0.03	−0.26	0.31	0.863	−0.05	−0.38	0.27	0.747
**Caring hours (ref 1**–**9 h/w)**^a^								
10 or + hrs/week	−0.23	−0.13	0.60	0.219	−0.28	−0.69	0.13	0.178
**Care recipient (ref parent)**^a^								
Other	−0.03	−0.43	0.37	0.874	0.06	−0.39	0.52	0.785
**Age group (non-carer ref)**								
Carers 10–17	0.11	−0.21	0.44	0.504	−0.17	−0.54	0.20	0.356
Carers 18–25	−0.44	−1.20	0.32	0.256	0.38	−0.48	1.24	0.384
**Gender (non-carer ref)**								
Male carers	−0.19	−0.63	0.24	0.092	0.20	−0.30	0.69	0.434
Female carers	0.32	−0.04	0.67	0.079	−0.41	−0.81	0.01	0.052
**Ethnicity (non-carer ref)**								
White carers	−0.11	−0.43	0.22	0.531	0.14	−0.23	0.50	0.460
Black carers	0.29	−0.78	1.35	0.593	−0.47	−1.69	0.75	0.453
Indian carers	−0.83	−2.42	0.75	0.303	0.79	−1.03	2.61	0.396
Pakistani/Bangladeshi carers	−0.47	−1.39	0.45	0.317	0.49	−0.55	1.54	0.353
Other Ethnic grp carers	−0.04	−1.45	1.37	0.955	0.09	−1.52	1.70	0.912
**Hhold income (non-carer ref)**								
Highest income quintile carers	−0.01	−0.05	0.04	0.824	−0.02	−0.05	0.05	0.948
Second quintile carers	0.01	−0.02	0.05	0.451	−0.01	−0.05	0.03	0.561
Third quintile carers	0.04	−0.01	0.07	0.148	−0.04	−0.08	0.01	0.117
Fourth quintile carers	−0.02	−0.07	0.02	0.286	0.03	−0.02	0.07	0.313
Lowest quintile carers	−0.02	−0.06	0.02	0.298	0.02	−0.02	0.07	0.302

a Interaction only tested among young carers.

## Discussion

Using a large, UK-representative panel study we found that life satisfaction declined upon becoming a young carer. This difference in life satisfaction between young carers and their peers began two years prior to reporting becoming a young carer and were sustained for three years after. Declines were more pronounced for young carers who provided 10+ hours of care per week, those from Black ethnic groups, and those from households in the lowest fifth of income. Changes in self-esteem and self-rated health were less clear-cut. A lower level of self-esteem and self-rated health was observed in the year prior to reporting being a young carer but no other differences were observed during or after transition to young caring. For self-rated health, females and those who cared for 10+ hours per week had lower levels of health two years prior to reporting being a young carer.

Previous research has found lower levels of life satisfaction in young carers relative to their peers.^20^, ^21^, ^22^ However, here we extended this to look at change during and after becoming a young carer—the first study to do so. This allows us to assess when differences in life satisfaction begin to emerge and how long-term any differences might be. We provide further empirical evidence for the first stage of caregiver identity theory,^11^ whereby the caring role has begun but the carer does not identify as such yet their health and wellbeing are starting to be affected. This was found in other studies applying the same analytic approach to adult carers.^9^^,^^10^ Our findings also suggest that the modest differences in life satisfaction persist in the medium-term. Similar to longitudinal work on adult carers, we find that the decline in life satisfaction is pronounced for carers who care more intensively^9^ and this is likely due to higher intensity caring placing particular constraints on time for social, leisure activities and education, as well as changes in family functioning and support^21^—activities and resources which are important for maintaining positive wellbeing.^23^^,^^24^ Furthermore, our study highlights additional inequalities for particular groups of UK young carers. We previously knew that young and young adult carers were more likely to come from more deprived backgrounds and be from ethnic minorities,^4^^,^^5^ but here we show that these play out as additional inequalities in life satisfaction changes over time. In the UK, like many other countries, there are ethnic and social inequalities in access to health and social care, plus heightened risks of significant illnesses, particularly mental illness.^25^^,^^26^ Consequently, it is possible that young carers from Black and low-income households shoulder more care.^27^

Unlike cross-sectional studies,^22^^,^^28^ we do not find differences in self-rated health over time between young carers and their peers. This might be because there is little variation in self-rated health categories in young people across time or because young carers use the health of the person they care for as their reference point. Similarly, we do not find differences for self-esteem despite cross-sectional studies^29^ demonstrating large differences in this between young carers and their peers. There is some evidence that self-esteem may be further downstream and affected by life satisfaction and it might therefore be that self-esteem changes are not observed within the timeframe of the present study.

We also found no gender differences. This is consistent with a lack of gender differences in young caring prevalence in the UK.^4^^,^^30^ Gender differences in caring begin to emerge in early adulthood,^5^^,^^10^ when traditional gender roles become more evident. Finally, the trajectories of health and wellbeing around becoming a young carer did not differ by age. This suggests that a broader policy age definition might be warranted, although it should be noted that the difference in the wording in the care questions between the youth and adult questionnaires make this challenging. Differences in the impacts of young caring should be tested in a dataset which has the same care question applied across ages.

The strengths of this study are the use of a large, UK-representative longitudinal study. This allowed us to take a UK-wide view of becoming a young carer and how it influences health and wellbeing up to 12 years before and after becoming a young carer—the longest-term study by far and one of very few longitudinal studies on young carers’ health and wellbeing. We also applied PSM to reduce some of the differences between young carers and non-carers which might confound associations with health and wellbeing.

Regarding limitations, the youth questionnaire young caring question is quite imprecise and does not match the question asked of people aged 16+. This may account for the higher percentage of young carers aged <18 years in our sample. We additionally explored the continuity in young carers identified across the two questionnaires (Appendix p. 21). 34.5% of young carers reported caring in more than one wave and 30% of those reported caring in both the youth and adult questionnaires. Regarding the youth questionnaire care question, the question imprecision raised concerns about young people reporting childcare/babysitting. We corrected for this as best as we could by only including young carers caring for a child if an adult in the household was also providing care. Second, while the UKHLS sample is large it is not large enough for a nuanced analysis of intersecting inequalities, for instance looking at differences by ethnicity and gender, nor do not have sufficient numbers to examine more finely grained ethnic groups. There is also no prior simulation study which guides the statistical power of our complex piecewise models, and it is possible that our work is underpowered, particularly when splitting down our analyses by ethnicity. Third, we do not have detailed information on the reason the care recipient requires care, what formal support they current receive, or what caring activities are undertaken by the carer. An analysis of these factors may reveal further differences in the impacts on health and wellbeing. Fourth, we used PSM to reduce confounding at baseline but our findings could be residually confounded or subject to time-varying confounding, for example young people with poorer health and wellbeing may be more likely to be carers. Fifth, life satisfaction and health outcomes were based on single item, subjective measures, although these are standard items using in many population surveys. Sixth, the young age of the sample limits the reliability of estimates 10+ years before becoming a carer, as these rely on extrapolation rather than actual data. While caution is needed further away from the caring transition, growth curve models remain a robust tool for comparing wellbeing trajectories over time between carers and non-carers. Seventh, there were slight differences in the included and excluded samples with those excluded being older, more likely from single parent households, and more likely to be from households from the lowest fifth of income (Appendix p. 22). Eight, we did not apply the survey weights to our initial analyses but sensitivity analyses with application of baseline cross-sectional weights for the main carer/non-carer analysis demonstrated no differences (Appendix p. 26 and pp. 16–18). Finally, we assumed that the first transition to young caring observed during the UKHLS survey period was the first transition in a young carers’ life. This may not be the case but is a problem for any study which looks at exposures within a survey period.

In summary, this study points to the importance of early identification and support for young carers to prevent impacts on wellbeing. This might be via health and social care services or education institutions where young carers will be in regular contact. Currently this support is piecemeal and relies on individual institutions recognising and providing dedicated support for young carers. This should be coupled with improving societal awareness of young caring, which is increasing in some, but not all,^3^ countries. The findings particularly highlight the importance of striving to reduce the care loads of young carers to prevent young people from providing excessive levels of care and one day becoming the care recipients of the future.

## Contributors

RL, AL, and AM conceptualised and designed the study. RL acquired funding for the study. AL analysed the data with input from BX and RL. AL and RL directly accessed and verified the underlying data reported in the manuscript. RL drafted the manuscript for publication. All authors approved the final draft and accept responsibility to submit for publication.

## Data sharing statement

UKHLS data are available to access and download via the UK Data Service: https://ukdataservice.ac.uk/ Information on the survey is available on the study website: https://www.understandingsociety.ac.uk/.

## Declaration of interests

We declare no competing interests.
