# Supplementary material for: Changes in life satisfaction, self-esteem, and self-rated health before, during, and after becoming a young carer in the UK: a longitudinal, propensity score analysis
Source: Lancet Reg Health Eur. 2024 Dec 20;50:101187. doi: 10.1016/j.lanepe.2024.101187 (PMC11726805; doi:10.1016/j.lanepe.2024.101187)
Supplement: Appendix [file mmc1.docx]

**Table of contents**

Appendix 1 – Availability of caring and outcome measurements across UKHLS waves

Appendix 2 - Description of study covariates

Appendix 3 - Checking the quality of the propensity score matching

Appendix 4 - Description of the analysis approach

Appendix 5 – Sample selection process

Appendix 6 – Predicted mean life satisfaction scores each year

Appendix 7 – Predicted mean self-esteem scores each year

Appendix 8 – Predicted mean probabilities of good/very good health each year

Appendix 9 -- Predicted mean life satisfaction per year - Sensitivity analysis: PSM with weights

Appendix 10 -- Predicted mean self-esteem scores per year - Sensitivity analysis: PSM with weights

Appendix 11 – Predicted mean probabilities of good self-rated health per year - PSM with weights

Appendix 12 -- Fitted Lines for Carers and Non-Carers Trajectories

Appendix 13 – Investigating continuity in young caring across youth and adult questionnaires

Appendix 14 – Comparing included and excluded samples20.

Appendix 15 – Sensitivity analyses applying survey weights

**Appendix 1 -- Availability of caring and outcome measurements across UKHLS waves**

| **Wave** | **1** | **2** | **3** | **4** | **5** | **6** | **7** | **8** | **9** | **10** | **11** | **12** | **13** |
| --- | --- | --- | --- | --- | --- | --- | --- | --- | --- | --- | --- | --- | --- |
| Caring (16+yrs) | x | x | x | x | x | x | x | x | x | x | x | x | X |
| Caring (10-15 yrs) |  |  | x |  | x |  | x |  | x |  | x |  | x |
| Life satisfaction (10-25 yrs) | x | x | x | x | x | x | x | x | x | x | x | x | X |
| Self-esteem (10-21 yrs) |  | x |  | x |  | x |  | x |  | x |  | x |  |
| Self-rated health (16+ yrs) | x | x | x | x | x | x | x | x | x | x | x | x | x |
| Self-rated health (10-15 yrs) |  | x |  | x |  | x |  | x |  | x |  | x |  |

**Appendix 2 -- Description of study covariates**

Age was banded into two groups indicating the policy definitions of young carers (<18 years) and young adult carers (18-25 years). Sex was self-reported in binary form (male/female). Ethnicity was categorised as: White, Black, Indian, Pakistani/Bangladeshi, or Other. Household income was reported as monthly net income, equilvalised using the OECD equivalence scale and split into quintiles. The number of natural, step- or adopted siblings in the household was categorised as 0, 1, 2, 3 or more. Parental occupational class was measured using the National Statistics Socio-Economic Classification. Categories were: Managerial/Professional, Intermediate, Routine and Not working. The highest class from either parent was used. The highest parental educational qualification obtained was categorised as Degree, Other Higher Qualification, Advanced (A) Level, General Certificate of Secondary Education (GCSE/CSE)-level, Other, or No qualifications. Household parental composition was categorised as two- or single-parent household. Urbanicity was either urban or rural and based on whether the participants’ home was located in a settlement with a population of 10,000 or more (“Urban”) or not (“Rural”) by the Office of National Statistics (ONS) Rural and Urban Classification of Output Areas 2001. The total number of waves the young person participated in was categorised as 2 to 13. All covariates were taken from baseline – the participants’ first observed wave.

**Appendix 3 – Checking the quality of the propensity score matching (PSM)**

The adequacy of the propensity score matching was checked using two methods. First, by checking the density plots of the propensity score for carers and non-carers before and after matching. Second, by checking the distributions of all covariates before and after matching. Three, by viewing the standardised differences to test the balance of covariates after PSM. All sets of outputs are shown below and show that the PSM has performed well, reducing much of the differences between carers and non-carers.

**Density plots**


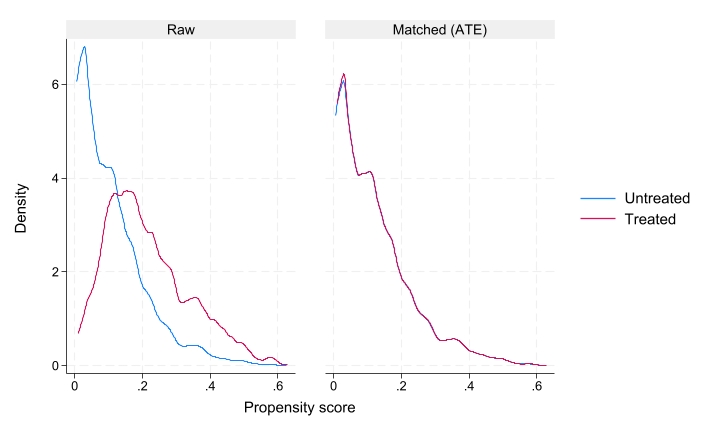


**Figure - Density plot for life satisfaction sample for the raw and matched untreated (non-carers) and treated (young carers)**

**
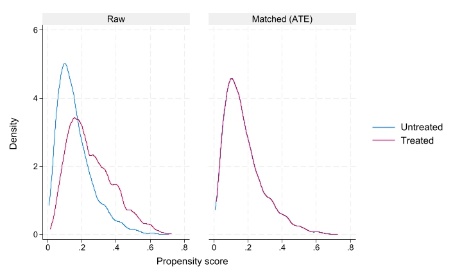
**

**Figure - Density plot for self-esteem sample for the raw and matched untreated (non-carers) and treated (young carers)**

**
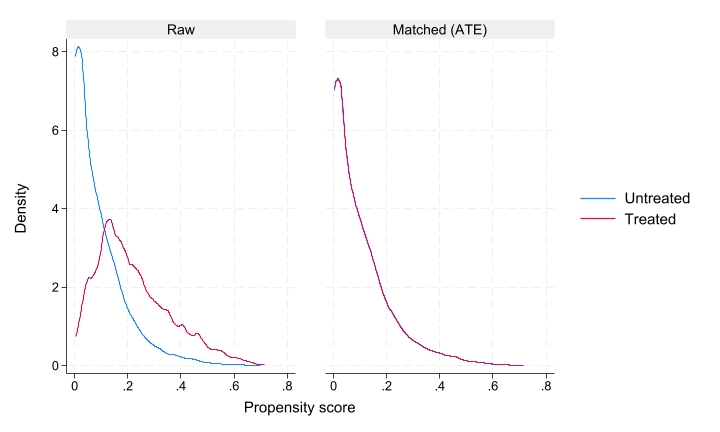
**

**Figure - Density plot for self-rated health sample for the raw and matched untreated (non-carers) and treated (young carers)**

**Observing changes in covariates after matching**

**Table - Comparing the balance of covariates in the life satisfaction sample pre- and post-PSM**

|  | **Pre PSM^a^** | | | **Post PSM^b^** | | | | | |  |
| --- | --- | --- | --- | --- | --- | --- | --- | --- | --- | --- |
|  | **Total** | **Non carers** | **Carers** | **Total ^c^** | | **Non carers** | | **Carers** | | |
| n | 19,022 | 16,622 | 2,400 | 4,424 | | 2,024  45.8 | | 2,400  54.3 | | |
| % |  | 87.4 | 12.6 |  |  |  |  |  |  |  |
|  | **%** | % | % | **%** | **n** | **%** | **n** | **%** | **n** | |
| **Gender** |  |  |  |  |  |  |  |  |  | |
| Male | 47.4 | 46.9 | 51.0 | 51.1 | 2261 | 51.3 | 1038 | 51.0 | 1223 | |
| Female | 52.6 | 53.1 | 49.0 | 48.9 | 2163 | 48.7 | 986 | 49.0 | 1177 | |
| **Age group** |  |  |  |  |  |  |  |  |  | |
| 10-17 | 67.0 | 63.6 | 90.5 | 92.3 | 4081 | 94.3 | 1908 | 90.5 | 2173 | |
| 18-25 | 33.0 | 36.4 | 9.5 | 7.8 | 343 | 5.7 | 116 | 9.5 | 227 | |
| **Caring hours (per week)** |  |  |  |  |  |  |  |  |  | |
| 1-9 hrs | 70.4 |  | 70.4 | 70.4 | 1,444 |  |  | 70.4 | 1,444 | |
| 10+ hrs | 29.6 |  | 29.6 | 29.6 | 608 |  |  | 29.6 | 608 | |
| **Care recipient** |  |  |  |  |  |  |  |  |  | |
| Parent | 69.9 |  | 69.9 | 69.9 | 1,327 |  |  | 69.9 | 1,327 | |
| Other | 30.1 |  | 30.1 | 30.1 | 572 |  |  | 30.1 | 572 | |
| **Ethnicity** |  |  |  |  |  |  |  |  |  | |
| White | 74.9 | 76 | 67.0 | 71.3 | 3156 | 76.4 | 1547 | 67.0 | 1609 | |
| Black | 7.5 | 7.5 | 8.0 | 7.2 | 318 | 6.3 | 127 | 8.0 | 191 | |
| Indian | 4.1 | 34 | 5.2 | 4.7 | 207 | 4.1 | 82 | 5.2 | 125 | |
| Pakistani/Bangladeshi | 8.5 | 7.5 | 15.5 | 12.7 | 563 | 9.4 | 191 | 15.5 | 372 | |
| Other | 5.0 | 5.1 | 4.3 | 4.1 | 180 | 3.8 | 77 | 4.3 | 103 | |
| **Urbanicity** |  |  |  |  |  |  |  |  |  | |
| Urban | 79.6 | 79.2 | 82.3 | 81.1 | 3575 | 79.1 | 1601 | 82.3 | 1974 | |
| Rural | 20.4 | 20.8 | 17.8 | 18.9 | 849 | 20.9 | 423 | 17.8 | 426 | |
| **Parental composition** |  |  |  |  |  |  |  |  |  | |
| Two parent household | 70.9 | 71.6 | 65.6 | 69.9 | 3090 | 74.9 | 1515 | 65.6 | 1575 | |
| Single parent household | 29.2 | 28.4 | 34.4 | 30.2 | 1334 | 25.2 | 509 | 34.4 | 825 | |
| **Number siblings in the hhold** |  |  |  |  |  |  |  |  |  | |
| None | 28.7 | 30.7 | 14.9 | 13.5 | 595 | 11.8 | 238 | 14.9 | 357 | |
| 1 | 36.7 | 36.3 | 39.2 | 42.9 | 1897 | 47.3 | 957 | 39.2 | 940 | |
| 2 | 21.0 | 20.6 | 24.4 | 25.0 | 1105 | 25.6 | 519 | 24.4 | 586 | |
| 3 or more | 14.0 | 12.4 | 21.5 | 18.7 | 827 | 15.3 | 310 | 21.5 | 517 | |
| Hhold income |  |  |  |  |  |  |  |  |  | |
| Highest quintile | 15.2 | 16.3 | 7.8 | 10.0 | 444 | 12.7 | 256 | 7.8 | 188 | |
| Second quintile | 19.8 | 20.7 | 13.5 | 16.3 | 723 | 19.7 | 398 | 13.5 | 325 | |
| Third quintile | 20.7 | 21 | 18.5 | 19.5 | 861 | 20.6 | 417 | 18.5 | 444 | |
| Fourth quintile | 21.6 | 20.6 | 28.7 | 27.0 | 1196 | 25.1 | 507 | 28.7 | 689 | |
| Lowest quintile | 22.7 | 21.4 | 31.4 | 27.1 | 1200 | 22.0 | 446 | 31.4 | 754 | |
| **Parental occupational class** |  |  |  |  |  |  |  |  |  | |
| Managerial/professional | 40.9 | 43.1 | 26.4 | 33.3 | 1472 | 41.4 | 735 | 26.4 | 634 | |
| Intermediate | 19.8 | 20.3 | 16.8 | 16.8 | 745 | 16.9 | 341 | 16.8 | 404 | |
| Routine | 23.9 | 23.7 | 25.1 | 23.5 | 1040 | 21.6 | 438 | 25.1 | 603 | |
| Not in paid work | 15.4 | 13.0 | 31.6 | 26.4 | 1167 | 20.2 | 368 | 31.6 | 759 | |
| **Parental education** |  |  |  |  |  |  |  |  |  | |
| Degree | 31.9 | 33.3 | 22.5 | 27.7 | 1226 | 33.9 | 686 | 22.5 | 540 | |
| Other Higher | 15.0 | 15.0 | 14.6 | 14.4 | 680 | 16.3 | 330 | 14.6 | 350 | |
| A-Level etc | 22.1 | 22.4 | 20.0 | 19.8 | 876 | 19.5 | 395 | 20.0 | 481 | |
| GSCE etc | 19.5 | 19.0 | 23.1 | 21.6 | 956 | 19.8 | 401 | 23.1 | 555 | |
| Other qualification | 5.5 | 5.1 | 8.6 | 7.0 | 308 | 5.0 | 101 | 8.6 | 207 | |
| No qualification | 6.0 | 5.3 | 11.1 | 8.5 | 378 | 5.5 | 108 | 11.1 | 267 | |

Abbreviations: PSM = propensity score matching

^a^Unweighted distributions, sample after applying inclusion/exclusion criteria, before PSM

^b^Unweighted distributions, sample after PSM

^c^Total analysis sample for carers vs non-carers and care intensity analysis

**Table - Comparing the balance of covariates in the self-esteem sample pre- and post-PSM**

|  | **Pre PSM^a^** | | | **Post PSM^b^** | | | | | |
| --- | --- | --- | --- | --- | --- | --- | --- | --- | --- |
|  | **Total** | **Non carers** | **Carers** | **Total^c^** | | **Non carers** | | **Carers** | |
| n | 8,052 | 6,638 | 1,414 | 2,453 | | 1,309 | | 1,414 | |
| % |  | 82.4 | 17.6 |  |  | 42.3 | | 57.6 | |
|  | **%** | % | % | **%** | **n** | **%** | **n** | **%** | **n** |
| **Gender** |  |  |  |  |  |  |  |  |  |
| Male | 48.6 | 48.2 | 50.8 | 50.5 | 1,238 | 50.1 | 520 | 50.8 | 718 |
| Female | 51.4 | 51.8 | 49.2 | 49.5 | 1,215 | 50.0 | 519 | 49.2 | 696 |
| **Age group** |  |  |  |  |  |  |  |  |  |
| 10-17 | 99.2 | 99.1 | 99.8 | 99.8 | 2,448 | 99.8 | 1,037 | 99.8 | 1,411 |
| 18-21 | 0.8 | 0.9 | 0.2 | 0.2 | 5 | 0.2 | 2 | 0.2 | 3 |
| **Caring hours (per week)** |  |  |  |  |  |  |  |  |  |
| 1-9 hrs | 77.5 |  | 77.5 | 77.5 | 916 |  |  | 77.5 | 916 |
| 10+ hrs | 22.5 |  | 22.5 | 22.5 | 266 |  |  | 22.5 | 266 |
| **Care recipient** |  |  |  |  |  |  |  |  |  |
| Parent | 72.9 |  | 72.9 | 72.9 | 771 |  |  | 72.9 | 771 |
| Other | 27.1 |  | 27.1 | 27.1 | 286 |  |  | 27.1 | 286 |
| **Ethnicity** |  |  |  |  |  |  |  |  |  |
| White | 76.2 | 77.7 | 69.2 | 73.2 | 1,795 | 78.5 | 816 | 69.2 | 979 |
| Black | 6.8 | 6.6 | 7.9 | 7.1 | 175 | 6.1 | 63 | 7.9 | 112 |
| Indian | 4.0 | 3.8 | 5.0 | 4.4 | 109 | 3.7 | 38 | 5.0 | 71 |
| Pakistani/Bangladeshi | 8.2 | 7.1 | 13.6 | 11.5 | 282 | 8.7 | 90 | 13.6 | 192 |
| Other | 4.8 | 4.9 | 4.2 | 3.8 | 92 | 3.1 | 32 | 4.2 | 60 |
| **Urbanicity** |  |  |  |  |  |  |  |  |  |
| Urban | 76.8 | 76.2 | 79.7 | 79.0 | 1,938 | 78.1 | 811 | 79.7 | 1,127 |
| Rural | 23.2 | 23.8 | 20.3 | 21.0 | 515 | 21.9 | 228 | 20.3 | 287 |
| **Parental composition** |  |  |  |  |  |  |  |  |  |
| Two parent household | 74.6 | 76.1 | 68.0 | 72.0 | 1,765 | 77.4 | 804 | 68.0 | 961 |
| Single parent household | 25.4 | 24.0 | 32.0 | 28.1 | 688 | 22.6 | 235 | 32.0 | 453 |
| **Number siblings in the hhold** |  |  |  |  |  |  |  |  |  |
| None | 13.6 | 13.8 | 12.8 | 13.1 | 320 | 13.4 | 139 | 12.8 | 181 |
| 1 | 44.1 | 45.0 | 39.6 | 42.0 | 1,031 | 45.3 | 471 | 39.6 | 560 |
| 2 | 26.2 | 26.2 | 26.5 | 26.6 | 652 | 26.7 | 277 | 26.5 | 375 |
| 3 or more | 16.1 | 15.0 | 21.1 | 18.3 | 450 | 14.6 | 152 | 21.1 | 298 |
| Hhold income |  |  |  |  |  |  |  |  |  |
| Highest quintile | 16.3 | 17.8 | 9.5 | 11.7 | 288 | 14.8 | 154 | 9.5 | 134 |
| Second quintile | 19.5 | 20.6 | 14.1 | 17.1 | 419 | 21.1 | 219 | 14.1 | 200 |
| Third quintile | 21.0 | 21.6 | 18.5 | 18.8 | 462 | 19.4 | 201 | 18.5 | 261 |
| Fourth quintile | 22.4 | 21.2 | 28.1 | 26.3 | 644 | 23.8 | 247 | 28.1 | 397 |
| Lowest quintile | 20.9 | 19.0 | 29.8 | 26.1 | 640 | 21.0 | 218 | 29.8 | 422 |
| **Parental occupational class** |  |  |  |  |  |  |  |  |  |
| Managerial/professional | 44.2 | 47.7 | 27.8 | 34.7 | 852 | 44.2 | 459 | 27.8 | 393 |
| Intermediate | 19.4 | 19.6 | 18.4 | 15.6 | 431 | 16.5 | 171 | 18.4 | 260 |
| Routine | 20.6 | 19.5 | 25.7 | 23.5 | 577 | 20.5 | 213 | 25.7 | 364 |
| Not in paid work | 15.8 | 13.2 | 28.1 | 24.2 | 593 | 18.9 | 196 | 28.1 | 397 |
| **Parental education** |  |  |  |  |  |  |  |  |  |
| Degree | 35.7 | 38.0 | 25.0 | 29.4 | 722 | 35.4 | 368 | 25.0 | 354 |
| Other Higher | 15.6 | 15.7 | 15.4 | 15.1 | 370 | 14.6 | 152 | 15.4 | 218 |
| A-Level etc | 19.7 | 19.8 | 19.2 | 19.1 | 468 | 18.9 | 196 | 19.2 | 272 |
| GSCE etc | 19.3 | 18.4 | 23.6 | 22.7 | 556 | 21.5 | 223 | 23.6 | 333 |
| Other qualification | 4.6 | 4.0 | 7.5 | 6.1 | 150 | 4.2 | 44 | 7.5 | 106 |
| No qualification | 5.1 | 4.2 | 9.3 | 7.6 | 187 | 5.4 | 56 | 9.3 | 131 |

Abbreviations: PSM = propensity score matching

^a^Unweighted distributions, sample after applying inclusion/exclusion criteria, before PSM

^b^Unweighted distributions, sample after PSM

^c^Total analysis sample for carers vs non-carers and care intensity analysis

**Table - Comparing the balance of covariates in the self-rated health sample pre- and post-PSM**

|  | **Pre PSM^a^** | | | **Post PSM^b^** | | | | | |  |
| --- | --- | --- | --- | --- | --- | --- | --- | --- | --- | --- |
|  | **Total** | **Non carers** | **Carers** | **Total^c^** | | **Non carers** | | **Carers** | | |
| n | 19,711 | 17,497 | 2,214 | 3,731 |  | 1,517 |  | 2,214 |  | |
| % |  | 88.8 | 11.2 |  |  | 40.6 |  | 59.3 |  | |
|  | **%** | % | % | **%** | **n** | **%** | **n** | **%** | **n** | |
| **Gender** |  |  |  |  |  |  |  |  |  | |
| Male | 48.2 | 47.9 | 50.1 | 49.8 | 1,859 | 49.5 | 751 | 50.1 | 1,108 | |
| Female | 51.8 | 52.1 | 49.9 | 50.2 | 1,872 | 50.5 | 766 | 50.0 | 1,106 | |
| **Age group** |  |  |  |  |  |  |  |  |  | |
| 10-17 | 61.3 | 58.3 | 85.1 | 87.5 | 3,265 | 91.0 | 1,380 | 85.1 | 1,885 | |
| 18-21 | 38.7 | 41.7 | 14.9 | 12.5 | 466 | 9.0 | 137 | 14.9 | 329 | |
| **Caring hours (per week)** |  |  |  |  |  |  |  |  |  | |
| 1-9 hrs | 68.6 |  | 68.6 | 68.6 | 1,299 |  |  | 68.6 | 1,299 | |
| 10+ hrs | 31.4 |  | 31.4 | 31.4 | 595 |  |  | 31.4 | 595 | |
| **Care recipient** |  |  |  |  |  |  |  |  |  | |
| Parent | 69.9 |  | 69.9 | 69.9 | 1,257 |  |  | 69.9 | 1,257 | |
| Other | 30.1 |  | 30.1 | 30.1 | 542 |  |  | 30.1 | 542 | |
| **Ethnicity** |  |  |  |  |  |  |  |  |  | |
| White | 73.9 | 74.9 | 65.5 | 69.7 | 2,601 | 75.9 | 1152 | 65.5 | 1,449 | |
| Black | 7.9 | 7.9 | 8.3 | 7.5 | 279 | 6.3 | 96 | 8.3 | 183 | |
| Indian | 4.3 | 4.1 | 5.7 | 5.0 | 187 | 4 | 61 | 5.7 | 126 | |
| Pakistani/Bangladeshi | 8.8 | 7.8 | 16.2 | 13.5 | 502 | 9.5 | 144 | 16.2 | 358 | |
| Other | 5.2 | 5.2 | 4.4 | 4.3 | 162 | 4.2 | 64 | 4.4 | 98 | |
| **Urbanicity** |  |  |  |  |  |  |  |  |  | |
| Urban | 80.0 | 79.6 | 82.7 | 81.1 | 3,024 | 78.7 | 1,194 | 82.7 | 1,830 | |
| Rural | 20.0 | 20.4 | 17.3 | 19.0 | 707 | 21.3 | 323 | 17.3 | 384 | |
| **Parental composition** |  |  |  |  |  |  |  |  |  | |
| Two parent household | 70.3 | 70.9 | 65.5 | 68.3 | 2,549 | 72.5 | 1100 | 65.5 | 1,449 | |
| Single parent household | 29.7 | 29.1 | 34.6 | 31.7 | 1,182 | 27.5 | 417 | 34.6 | 765 | |
| **Number siblings in the hhold** |  |  |  |  |  |  |  |  |  | |
| None | 30.6 | 32.5 | 15.6 | 15.3 | 570 | 14.8 | 224 | 15.6 | 346 | |
| 1 | 35.9 | 35.6 | 38.9 | 41.6 | 1,552 | 45.5 | 690 | 38.9 | 862 | |
| 2 | 20.4 | 19.8 | 24.9 | 25.4 | 947 | 26.1 | 396 | 24.9 | 551 | |
| 3 or more | 13.1 | 12.1 | 20.6 | 17.7 | 662 | 13.7 | 207 | 20.6 | 455 | |
| Hhold income |  |  |  |  |  |  |  |  |  | |
| Highest | 15.4 | 16.3 | 8.4 | 9.9 | 368 | 12.1 | 183 | 8.4 | 185 | |
| Second quintile | 20 | 20.9 | 13.4 | 16.4 | 612 | 20.8 | 315 | 13.4 | 297 | |
| Third quintile | 20.6 | 20.9 | 18.1 | 19.1 | 713 | 20.6 | 312 | 18.1 | 401 | |
| Fourth quintile | 21.1 | 20.1 | 28.4 | 27.0 | 1,008 | 25.0 | 379 | 28.4 | 629 | |
| Lowest | 22.9 | 21.8 | 31.7 | 27.6 | 1,030 | 21.6 | 328 | 31.7 | 702 | |
| **Parental occupational class** |  |  |  |  |  |  |  |  |  | |
| Managerial/professional | 40.6 | 45.2 | 26.5 | 32.4 | 1,207 | 40.9 | 621 | 26.5 | 586 | |
| Intermediate | 19.7 | 20.2 | 16.3 | 16.6 | 618 | 17.0 | 258 | 16.3 | 360 | |
| Routine | 24.1 | 24.1 | 24 | 23.6 | 882 | 23.1 | 350 | 24.0 | 532 | |
| Not in paid work | 15.6 | 13.2 | 33.2 | 27.5 | 1,024 | 19.0 | 288 | 33.2 | 736 | |
| **Parental education** |  |  |  |  |  |  |  |  |  | |
| Degree | 31.6 | 32.7 | 23.2 | 27.2 | 1,016 | 33.1 | 502 | 23.2 | 514 | |
| Other Higher | 14.9 | 15.0 | 14.3 | 14.8 | 552 | 15.6 | 237 | 14.2 | 315 | |
| A-Level etc | 22.0 | 22.3 | 19.7 | 20.1 | 749 | 20.7 | 314 | 19.7 | 435 | |
| GSCE etc | 19.6 | 19.1 | 23.0 | 21.7 | 811 | 19.9 | 301 | 23.0 | 510 | |
| Other qualification | 5.6 | 5.2 | 8.5 | 7.0 | 262 | 4.9 | 75 | 8.5 | 187 | |
| No qualification | 6.3 | 5.7 | 11.4 | 9.1 | 341 | 5.8 | 88 | 11.4 | 253 | |

Abbreviations: PSM = propensity score matching

^a^Unweighted distributions, sample after applying inclusion/exclusion criteria, before PSM

^b^Unweighted distributions, sample after PSM

^c^Total analysis sample for carers vs non-carers and care intensity analysis

**Table – Standardised differences to test balance of covariates after PSM – life satisfaction sample**

|  | **PSM without weights** | **PSM with weights** |
| --- | --- | --- |
| ethnicity | 0.02 | 0.001 |
| urbanicity | 0.07 | 0.08 |
| numbers of siblings | 0.02 | 0.02 |
| parental composition | 0.1 | 0.1 |
| household income | 0.1 | 0.1 |
| occupational class | 0.2 | 0.2 |
| parental qualifications | 0.2 | 0.2 |
| number of waves observed | 0.02 | 0.01 |

**Table – Standardised differences to test balance of covariates after PSM – self esteem sample**

|  | **PSM without weights** | **PSM with weights** |
| --- | --- | --- |
| ethnicity | 0.06 | 0.03 |
| urbanicity | 0.04 | 0.08 |
| numbers of siblings | 0.004 | 0.05 |
| parental composition | 0.1 | 0.1 |
| household income | 0.1 | 0.2 |
| occupational class | 0.2 | 0.3 |
| parental qualifications | 0.1 | 0.2 |
| number of waves observed | 0.02 | 0.01 |

**Table – Standardised differences to test balance of covariates after PSM – self-rated health sample**

|  | **PSM without weights** | **PSM with weights** |
| --- | --- | --- |
| ethnicity | 0.01 | 0.03 |
| urbanicity | 0.1 | 0.05 |
| numbers of siblings | 0.001 | 0.03 |
| parental composition | 0.1 | 0.1 |
| household income | 0.1 | 0.1 |
| occupational class | 0.3 | 0.3 |
| parental qualifications | 0.2 | 0.2 |
| number of waves observed | 0.02 | 0.04 |

**Appendix 4-- Description of the analysis approach**

To investigate wellbeing before, during, and after the onset of young caring the analyses followed these steps:

1. Piecewise linear regression: A piecewise linear regression model was constructed by dividing the time variable into three segments, representing time before, at transition and after the transition to becoming a young carer.
2. Multilevel mixed-effects linear regression: A multilevel mixed-effects linear regression model was used to analyse wellbeing over time, allowing for random intercepts at both the household and individual levels. The young carer status variable was included in the model, along with interaction terms to assess its effects on different segments of the time scale.
3. Graphing marginal effects: Separate regression were run for carers and non-carers. The marginal effects of time on wellbeing were plotted, illustrating changes in wellbeing before, during and after the onset of young caring for both groups.
4. Testing significance: The final model includes interaction terms between young carer status and the time segments, allowing us to test the significance of differences in wellbeing trajectories between carers and non-carers.

**Appendix 5 – Sample selection process**

**Figure – Sample selection process for life satisfaction analyses**

**Aged 10-25 and answered yes/no to carer Q**

N=27,678 (4,484 carers, 23,194 non-carers)

**Select carers who have ≥1 life satisfaction measure before & after young carer transition**

N=19,122 (2,500 carers, 16,622 non-carers)

**Exclude carers who have missing covariate data**

N=19,022 (2,400 carers, 16,622 non-carers)

| **Apply propensity score matching (PSM), 1:2 matching^a^** | | | | |
| --- | --- | --- | --- | --- |
|  | **Carer**  **Analysis^b^** | **Age**  **analysis** | **Ethnicity**  **analysis** | **Hhold income**  **analysis** |
| Carers | 2,400 | 2,400 | 2,400 | 2,400 |
| Non-carers | 3,657 | 4,179 | 3,684 | 3,675 |
| Total | 6,057 | 6,579 | 6,084 | 6,075 |

| **Only select non-carers who have ≥1 life satisfaction measure before & after matched caring transition**  **Final analytic samples** | | | | |
| --- | --- | --- | --- | --- |
|  | **Carer**  **Analysis^b^** | **Age**  **analysis** | **Ethnicity**  **analysis** | **Hhold income**  **analysis** |
| Carers | 2,400 | 2,400 | 2,400 | 2,400 |
| Non-carers | 2,024 | 1,502 | 2,103 | 2,206 |
| Total | 4,424 | 3,902 | 4,503 | 4,606 |

^a^PSM run separately for each inequality analysis removing that particular variable from the PSM e.g. ethnicity omitted from PSM for ethnicity inequality analysis but included in all other PSMs

^b^Caring hours (n=2052; 1-9 hrs per week = 1444; 10+ hrs per week = 608) and care recipient (n=1899; parent = 1327; other = 572) analyses run for carers only

**Figure – Sample selection process for self-esteem analyses**

**Aged 10-21 and answered yes/no to carer Q^a^**

N=21,842 (4,124 carers, 17,718 non-carers)

**Select carers who have ≥1 self esteem measure before & after young carer transition**

N=8,082 (1,444 carers, 6,638 non-carers)

**Exclude carers who have missing covariate data**

N=8,052 (1,414 carers, 6,638 non-carers)

| **Apply propensity score matching (PSM), 1:2 matching^b^** | | | | |
| --- | --- | --- | --- | --- |
|  | **Carer**  **Analysis^b^** | **Age**  **analysis** | **Ethnicity**  **analysis** | **Hhold income**  **analysis** |
| Carers | 2,070 | 2,315 | 2,038 | 2,147 |
| Non-carers | 1,414 | 1,414 | 1,414 | 1,414 |
| Total | 3,484 | 3,729 | 3,452 | 3,561 |

| **Only select non-carers who have ≥1 life satisfaction measure before & after matched caring transition**  **Final analytic samples** | | | | |
| --- | --- | --- | --- | --- |
|  | **Carer**  **Analysis^b^** | **Age**  **analysis** | **Ethnicity**  **analysis** | **Hhold income**  **analysis** |
| Carers | 1,414 | 1,414 | 1,414 | 1,414 |
| Non-carers | 1,039 | 906 | 1,094 | 1,137 |
| Total | 2,453 | 2,320 | 2,508 | 2,551 |

^a^Self-esteem only asked of young people aged 10-21 years

^b^PSM run separately for each inequality analysis removing that particular variable from the PSM e.g. ethnicity omitted from PSM for ethnicity inequality analysis but included in all other PSMs

^b^Caring hours (n=1182; 1-9 hrs per week = 916; 10+ hrs per week = 266) and care recipient (n=1057; parent = 771; other = 286) analyses run for carers only

**Figure – Sample selection process for self-rated health analyses**

**Aged 10-25 and answered yes/no to carer Q**

N=27,678 (4,484 carers, 23,194 non-carers)

**Select carers who have ≥1 self-rated measure before & after young carer transition**

N=19,818 (2,321 carers, 17,497 non-carers)

**Exclude carers who have missing covariate data**

N=19,711 (2,214 carers, 17,497 non-carers)

| **Apply propensity score matching (PSM), 1:2 matching^a^** | | | | |
| --- | --- | --- | --- | --- |
|  | **Carer**  **Analysis^b^** | **Age**  **analysis** | **Ethnicity**  **analysis** | **Hhold income**  **analysis** |
| Carers | 2,214 | 2,214 | 2,214 | 2,214 |
| Non-carers | 3,392 | 3,696 | 3,442 | 3,496 |
| Total | 5,606 | 5,910 | 5,656 | 5,710 |

| **Only select non-carers who have ≥1 life satisfaction measure before & after matched caring transition**  **Final analytic samples** | | | | |
| --- | --- | --- | --- | --- |
|  | **Carer**  **Analysis^b^** | **Age**  **analysis** | **Ethnicity**  **analysis** | **Hhold income**  **analysis** |
| Carers | 2,214 | 2,214 | 2,214 | 2,214 |
| Non-carers | 1,517 | 1,262 | 1,632 | 1,621 |
| Total | 3,731 | 3,476 | 3,846 | 3,835 |

^a^PSM run separately for each inequality analysis removing that particular variable from the PSM e.g. ethnicity omitted from PSM for ethnicity inequality analysis but included in all other PSMs

^b^Caring hours (n=1894; 1-9 hrs per week = 1299; 10+ hrs per week = 595) and care recipient (n=1799; parent = 1257; other = 542) analyses run for carers only

**Appendix 6 - Predicted mean life satisfaction per year**

| **Carers** | | | | | | **Non-Carers** | | | | | |
| --- | --- | --- | --- | --- | --- | --- | --- | --- | --- | --- | --- |
| **Year** | **Mean (AME)** | | | **95%CI** | | **Year** | **Mean (AME)** | | | **95%CI** | |
| -10 | | 6.80 | 6.13 | | 7.24 | -10 | | 6.57 | 5.88 | | 6.72 |
| -9 | | 6.10 | 5.68 | | 6.48 | -9 | | 6.40 | 5.94 | | 6.57 |
| -8 | | 6.13 | 5.76 | | 6.42 | -8 | | 6.22 | 5.9 | | 6.41 |
| -7 | | 6.28 | 6.03 | | 6.54 | -7 | | 6.28 | 5.91 | | 6.32 |
| -6 | | 5.88 | 5.67 | | 6.11 | -6 | | 6.06 | 5.83 | | 6.18 |
| -5 | | 6.01 | 5.83 | | 6.18 | -5 | | 6.06 | 5.9 | | 6.1 |
| -4 | | 5.80 | 5.68 | | 5.94 | -4 | | 5.98 | 5.84 | | 6.07 |
| -3 | | 5.73 | 5.64 | | 5.85 | -3 | | 5.92 | 5.84 | | 6.03 |
| -2 | | 5.70 | 5.63 | | 5.79 | -2 | | 5.86 | 5.78 | | 5.94 |
| -1 | | 5.63 | 5.57 | | 5.71 | -1 | | 5.80 | 5.78 | | 5.91 |
| 0 | | 5.49 | 5.44 | | 5.56 | 0 | | 5.67 | 5.64 | | 5.77 |
| 1 | | 5.36 | 5.29 | | 5.43 | 1 | | 5.59 | 5.55 | | 5.68 |
| 2 | | 5.26 | 5.18 | | 5.33 | 2 | | 5.40 | 5.35 | | 5.49 |
| 3 | | 5.17 | 5.09 | | 5.25 | 3 | | 5.35 | 5.3 | | 5.45 |
| 4 | | 5.11 | 5.02 | | 5.2 | 4 | | 5.26 | 5.15 | | 5.31 |
| 5 | | 5.07 | 4.98 | | 5.16 | 5 | | 5.20 | 5.13 | | 5.31 |
| 6 | | 4.95 | 4.85 | | 5.05 | 6 | | 5.07 | 4.97 | | 5.17 |
| 7 | | 4.86 | 4.75 | | 4.97 | 7 | | 4.98 | 4.82 | | 5.04 |
| 8 | | 4.83 | 4.72 | | 4.95 | 8 | | 5.02 | 4.88 | | 5.12 |
| 9 | | 4.77 | 4.62 | | 4.92 | 9 | | 5.06 | 4.73 | | 5.03 |
| 10 | | 4.72 | 4.56 | | 4.88 | 10 | | 5.00 | 4.65 | | 5.01 |
| 11 | | 4.53 | 4.11 | | 4.94 | 11 | | 4.77 | 4.59 | | 5.20 |

Abbreviations: AME = average marginal effect

**Appendix 7 – Predicted mean self-esteem scores per year**

| **Carers** | | | | | | **Non-Carers** | | | | | |
| --- | --- | --- | --- | --- | --- | --- | --- | --- | --- | --- | --- |
| **Year** | **Mean (AME)** | | | **95%CI** | | **Year** | | **Mean (AME)** | | **95%CI** | |
| -7 | | 26.0 | 24.2 | | 27.7 | -7 | 26.6 | | 24.9 | | 28.4 |
| -6 | | 25.4 | 24.3 | | 26.5 | -6 | 25.8 | | 24.3 | | 27.3 |
| -5 | | 25.7 | 24.7 | | 26.7 | -5 | 25.3 | | 24.4 | | 26.2 |
| -4 | | 24.7 | 23.9 | | 25.5 | -4 | 25.5 | | 24.9 | | 26.2 |
| -3 | | 24.9 | 24.5 | | 25.4 | -3 | 24.8 | | 24.3 | | 25.3 |
| -2 | | 24.8 | 24.3 | | 25.5 | -2 | 25.6 | | 25.2 | | 26.0 |
| -1 | | 24.6 | 24.3 | | 24.8 | -1 | 24.8 | | 24.6 | | 25.1 |
| 0 | | 24.0 | 24.5 | | 24.5 | 0 | 25.0 | | 24.4 | | 25.4 |
| 1 | | 24.3 | 24.0 | | 24.5 | 1 | 24.7 | | 23.8 | | 25.0 |
| 2 | | 23.6 | 22.9 | | 24.3 | 2 | 24.2 | | 23.6 | | 24.6 |
| 3 | | 23.8 | 23.6 | | 24.1 | 3 | 24.0 | | 23.5 | | 24.3 |
| 4 | | 23.9 | 23.2 | | 24.7 | 4 | 24.0 | | 23.5 | | 24.5 |
| 5 | | 23.8 | 23.4 | | 24.1 | 5 | 24.0 | | 23.6 | | 24.5 |
| 6 | | 23.6 | 22.7 | | 24.5 | 6 | 23.4 | | 22.7 | | 24.1 |
| 7 | | 22.9 | 22.5 | | 23.4 | 7 | 23.1 | | 22.5 | | 23.8 |
| 8 | | 23.1 | 21.6 | | 24.5 | 8 | 24.0 | | 22.8 | | 25.2 |
| 9 | | 22.1 | 21.4 | | 22.9 | 9 | 23.2 | | 22.1 | | 24.4 |

Abbreviations: AME = Average Marginal Effect

**Appendix 8 – Predicted mean probabilities of good self-rated health per year**

| **Carers** | | | | | | **Non-Carers** | | | | | | |
| --- | --- | --- | --- | --- | --- | --- | --- | --- | --- | --- | --- | --- |
| **Year** | **Mean (APP)** | | | **95%CI** | | | **Year** | **Mean (APP)** | | | **95%CI** | |
| -9 | | 0.91 | 0.84 | | 0.99 | | -9 | | 0.87 | 0.78 | | 0.97 |
| -8 | | 0.99 | 0.97 | | 1.00 | | -8 | | 0.95 | 0.90 | | 0.99 |
| -7 | | 0.90 | 0.85 | | 0.95 | | -7 | | 0.95 | 0.92 | | 0.99 |
| -6 | | 0.91 | 0.87 | | 0.95 | | -6 | | 0.96 | 0.93 | | 0.99 |
| -5 | | 0.94 | 0.91 | | 0.96 | | -5 | | 0.95 | 0.92 | | 0.97 |
| -4 | | 0.92 | 0.89 | | 0.94 | | -4 | | 0.95 | 0.93 | | 0.97 |
| -3 | | 0.91 | 0.89 | | 0.93 | | -3 | | 0.93 | 0.91 | | 0.95 |
| -2 | | 0.91 | 0.89 | | 0.93 | | -2 | | 0.93 | 0.91 | | 0.94 |
| -1 | | 0.91 | 0.90 | | 0.92 | | -1 | | 0.93 | 0.91 | | 0.94 |
| 0 | | 0.87 | 0.86 | | 0.89 | | 0 | | 0.93 | 0.91 | | 0.94 |
| 1 | | 0.89 | 0.87 | | 0.90 | | 1 | | 0.90 | 0.89 | | 0.92 |
| 2 | | 0.88 | 0.86 | | 0.90 | | 2 | | 0.92 | 0.91 | | 0.94 |
| 3 | | 0.88 | 0.86 | | 0.89 | | 3 | | 0.92 | 0.90 | | 0.94 |
| 4 | | 0.86 | 0.84 | | 0.88 | | 4 | | 0.91 | 0.89 | | 0.93 |
| 5 | | 0.87 | 0.85 | | 0.89 | | 5 | | 0.90 | 0.87 | | 0.93 |
| 6 | | 0.83 | 0.81 | | 0.86 | | 6 | | 0.89 | 0.86 | | 0.92 |
| 7 | | 0.82 | 0.79 | | 0.85 | | 7 | | 0.88 | 0.84 | | 0.91 |
| 8 | | 0.83 | 0.79 | | 0.86 | | 8 | | 0.86 | 0.82 | | 0.90 |
| 9 | | 0.78 | 0.80 | | 0.82 | | 9 | | 0.88 | 0.84 | | 0.93 |
| 10 | | 0.79 | 0.74 | | 0.84 | | 10 | | 0.85 | 0.78 | | 0.92 |

Abbreviations: APP = average predicted probabilities

**Appendix 9 -- Predicted mean life satisfaction per year**

**-- Sensitivity analysis: PSM with weights**

| **Carers** | | | | | | **Non-Carers** | | | | | |
| --- | --- | --- | --- | --- | --- | --- | --- | --- | --- | --- | --- |
| **Year** | **Mean (AME)** | | | **95%CI** | | **Year** | **Mean (AME)** | | | **95%CI** | |
| -10 | | 6.83 | 6.39 | | 7.27 | -10 | | 6.47 | 5.97 | | 6.98 |
| -9 | | 6.13 | 5.77 | | 6.48 | -9 | | 6.25 | 5.85 | | 6.66 |
| -8 | | 6.15 | 5.83 | | 6.46 | -8 | | 6.56 | 6.24 | | 6.88 |
| -7 | | 6.29 | 6.05 | | 6.53 | -7 | | 6.32 | 6.07 | | 6.57 |
| -6 | | 5.97 | 5.76 | | 6.19 | -6 | | 6.25 | 6.06 | | 6.44 |
| -5 | | 6.07 | 5.89 | | 6.24 | -5 | | 6.26 | 6.11 | | 6.40 |
| -4 | | 5.87 | 5.74 | | 6.00 | -4 | | 6.01 | 5.90 | | 6.13 |
| -3 | | 5.81 | 5.70 | | 5.92 | -3 | | 6.05 | 5.95 | | 6.14 |
| -2 | | 5.73 | 5.65 | | 5.81 | -2 | | 5.93 | 5.86 | | 6.01 |
| -1 | | 5.69 | 5.63 | | 5.76 | -1 | | 5.91 | 5.84 | | 5.97 |
| 0 | | 5.54 | 5.48 | | 5.60 | 0 | | 5.77 | 5.71 | | 5.83 |
| 1 | | 5.43 | 5.36 | | 5.51 | 1 | | 5.60 | 5.53 | | 5.67 |
| 2 | | 5.32 | 5.24 | | 5.40 | 2 | | 5.49 | 5.41 | | 5.56 |
| 3 | | 5.23 | 5.14 | | 5.31 | 3 | | 5.43 | 5.35 | | 5.50 |
| 4 | | 5.15 | 5.07 | | 5.24 | 4 | | 5.30 | 5.22 | | 5.38 |
| 5 | | 5.12 | 5.02 | | 5.21 | 5 | | 5.24 | 5.15 | | 5.33 |
| 6 | | 5.01 | 4.91 | | 5.11 | 6 | | 5.15 | 5.05 | | 5.24 |
| 7 | | 4.90 | 4.79 | | 5.01 | 7 | | 5.07 | 4.96 | | 5.17 |
| 8 | | 4.88 | 4.76 | | 5.00 | 8 | | 5.04 | 4.92 | | 5.16 |
| 9 | | 4.81 | 4.66 | | 4.96 | 9 | | 5.08 | 4.94 | | 5.22 |
| 10 | | 4.77 | 4.61 | | 4.93 | 10 | | 4.91 | 4.74 | | 5.09 |
| 11 | | 4.57 | 4.16 | | 4.98 | 11 | | 4.74 | 4.46 | | 5.02 |

Abbreviations: AME = average marginal effect

**Appendix 10 – Predicted mean self-esteem scores per year**

**-- Sensitivity analysis: PSM with weights**

| **Carers** | | | | | | **Non-Carers** | | | | | |
| --- | --- | --- | --- | --- | --- | --- | --- | --- | --- | --- | --- |
| **Year** | **Mean (AME)** | | | **95%CI** | | **Year** | | **Mean (AME)** | | **95%CI** | |
| -7 | | 26.1 | 24.3 | | 27.8 | -7 | 25.2 | | 24.3 | | 26.2 |
| -6 | | 25.3 | 24.2 | | 26.4 | -6 | 25.3 | | 24.5 | | 26.1 |
| -5 | | 25.9 | 24.9 | | 26.9 | -5 | 25.6 | | 25.0 | | 26.3 |
| -4 | | 24.8 | 24.1 | | 25.6 | -4 | 25.4 | | 24.9 | | 25.8 |
| -3 | | 25.0 | 24.5 | | 25.4 | -3 | 25.4 | | 25.0 | | 25.8 |
| -2 | | 24.9 | 24.2 | | 25.5 | -2 | 25.2 | | 24.9 | | 25.6 |
| -1 | | 24.6 | 24.3 | | 24.8 | -1 | 25.0 | | 24.7 | | 25.3 |
| 0 | | 24.0 | 23.4 | | 24.5 | 0 | 24.8 | | 24.5 | | 25.0 |
| 1 | | 24.3 | 24.0 | | 24.5 | 1 | 24.7 | | 24.4 | | 25.0 |
| 2 | | 23.6 | 23.0 | | 24.3 | 2 | 24.2 | | 23.9 | | 24.5 |
| 3 | | 23.8 | 23.6 | | 24.1 | 3 | 24.5 | | 24.2 | | 24.8 |
| 4 | | 23.9 | 23.1 | | 24.7 | 4 | 24.0 | | 23.7 | | 24.3 |
| 5 | | 23.8 | 23.5 | | 24.1 | 5 | 24.0 | | 23.7 | | 24.4 |
| 6 | | 23.6 | 22.7 | | 24.5 | 6 | 24.2 | | 23.8 | | 24.5 |
| 7 | | 22.9 | 22.5 | | 23.4 | 7 | 23.9 | | 23.5 | | 24.3 |
| 8 | | 22.6 | 21.1 | | 24.1 | 8 | 24.1 | | 23.5 | | 24.6 |
| 9 | | 22.2 | 21.4 | | 22.9 | 9 | 23.8 | | 23.2 | | 24.4 |

Abbreviations: AME = Average Marginal Effect

**Appendix 11 – Predicted mean probabilities of good self-rated health per year**

**-- PSM with weights**

| **Carers** | | | | | | **Non-Carers** | | | | | | |
| --- | --- | --- | --- | --- | --- | --- | --- | --- | --- | --- | --- | --- |
| **Year** | **Mean (APP)** | | | **95%CI** | | | **Year** | **Mean (APP)** | | | **95%CI** | |
| -9 | | 0.92 | 0.85 | | 0.99 | | -9 | | 0.85 | 0.59 | | 1.11 |
| -8 | | 0.98 | 0.96 | | 0.99 | | -8 | | 0.95 | 0.89 | | 1.00 |
| -7 | | 0.90 | 0.85 | | 0.96 | | -7 | | 0.95 | 0.91 | | 0.99 |
| -6 | | 0.92 | 0.87 | | 0.96 | | -6 | | 0.95 | 0.91 | | 1.00 |
| -5 | | 0.93 | 0.90 | | 0.96 | | -5 | | 0.96 | 0.93 | | 0.98 |
| -4 | | 0.93 | 0.90 | | 0.95 | | -4 | | 0.97 | 0.96 | | 0.99 |
| -3 | | 0.92 | 0.90 | | 0.94 | | -3 | | 0.94 | 0.92 | | 0.96 |
| -2 | | 0.91 | 0.88 | | 0.93 | | -2 | | 0.95 | 0.93 | | 0.97 |
| -1 | | 0.92 | 0.90 | | 0.93 | | -1 | | 0.94 | 0.93 | | 0.96 |
| 0 | | 0.88 | 0.86 | | 0.90 | | 0 | | 0.92 | 0.90 | | 0.94 |
| 1 | | 0.90 | 0.89 | | 0.92 | | 1 | | 0.92 | 0.90 | | 0.94 |
| 2 | | 0.89 | 0.87 | | 0.91 | | 2 | | 0.92 | 0.90 | | 0.94 |
| 3 | | 0.89 | 0.87 | | 0.91 | | 3 | | 0.90 | 0.87 | | 0.92 |
| 4 | | 0.87 | 0.85 | | 0.89 | | 4 | | 0.89 | 0.87 | | 0.92 |
| 5 | | 0.88 | 0.86 | | 0.91 | | 5 | | 0.89 | 0.86 | | 0.92 |
| 6 | | 0.85 | 0.82 | | 0.87 | | 6 | | 0.89 | 0.86 | | 0.92 |
| 7 | | 0.83 | 0.80 | | 0.86 | | 7 | | 0.89 | 0.86 | | 0.92 |
| 8 | | 0.85 | 0.82 | | 0.88 | | 8 | | 0.89 | 0.85 | | 0.93 |
| 9 | | 0.80 | 0.75 | | 0.84 | | 9 | | 0.84 | 0.78 | | 0.89 |
| 10 | | 0.81 | 0.76 | | 0.86 | | 10 | | 0.88 | 0.81 | | 0.94 |

Abbreviations: APP = average predicted probabilities

**Appendix 12 – Fitted Lines for Carers and Non-Carers Trajectories**

Figure – Life satisfaction trajectories before, during and after becoming a young carer


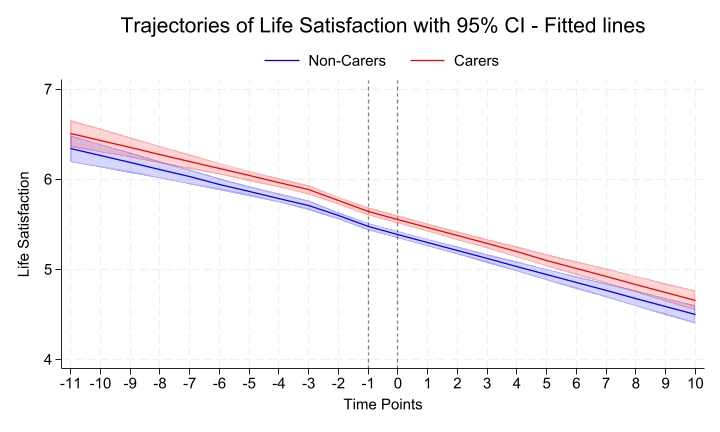


Figure – Self-esteem trajectories before, during and after becoming a young carer


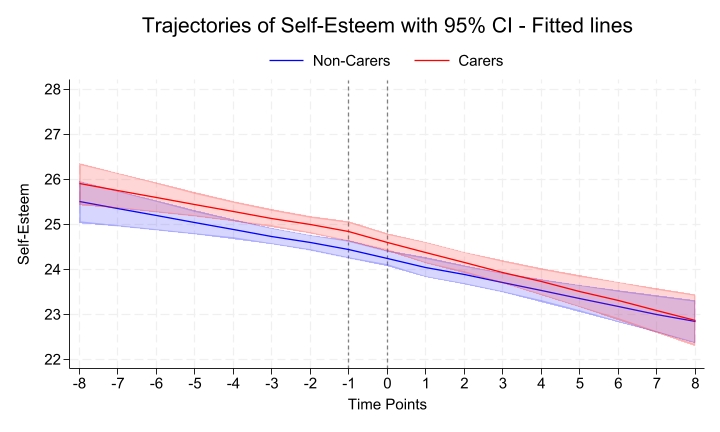


Figure – Self-rated health trajectories before, during and after becoming a young carer


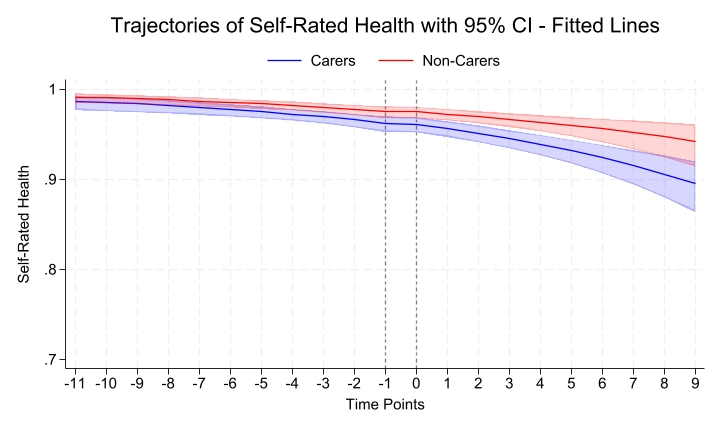


**Appendix 13 - Investigating continuity in young caring across youth and adult questionnaires**

Table – Count of waves in which young carers reported providing care in the life satisfaction sample

| **Number of waves** | **n** | **%** |
| --- | --- | --- |
| 1 | 1572 | 65.5 |
| 2 | 477 | 19.9 |
| 3 | 164 | 6.8 |
| 4 | 84 | 3.5 |
| 5 | 53 | 2.2 |
| 6 | 24 | 1.0 |
| 7 | 11 | 0.5 |
| 8 | 9 | 0.4 |
| 9 | 4 | 0.2 |
| 10 | 2 | 0.1 |

Table – Reporting as a young carer on both the youth and adult questionnaires in the life satisfaction sample

|  | **n** | **%** |
| --- | --- | --- |
| report caring only youth or adult quest | 584 | 70.5 |
| report caring on both youth and adult quest | 244 | 29.5 |

**Appendix 14 – Comparing included and excluded samples**

Table – Descriptive comparison of individuals excluded from the analysis and the analytic sample prior to PS for the life satisfaction sample

|  | **Pre PSM** | | | | | |
| --- | --- | --- | --- | --- | --- | --- |
|  | **Individuals Included in the PSM** | | | **Individuals Excluded from the PSM** | | |
|  | **total** | **Non carers** | **Carers** | **total** | **Non carers** | **Carers** |
| n | **19,022** | **16,622** | **2,400** | **8,656** | **6,572** | **2,084** |
|  | **%** | **%** | **%** | **%** | **%** | **%** |
| **Gender** |  |  |  |  |  |  |
| Male | 47.4 | 46.9 | 51.0 | 52.0 | 52.6 | 50.2 |
| Female | 52.6 | 53.1 | 49.0 | 48.0 | 47.4 | 49.8 |
| **Age first observed** |  |  |  |  |  |  |
| 10 to 17 | 67.0 | 63.6 | 90.5 | 39.1 | 30.7 | 65.8 |
| 18 to 25 | 33.0 | 36.4 | 9.5 | 60.9 | 69.3 | 34.2 |
| **Ethnicity** |  |  |  |  |  |  |
| White | 74.9 | 76.0 | 67.0 | 65.6 | 68.1 | 57.7 |
| Black | 7.5 | 7.5 | 8.0 | 9.9 | 9.8 | 10.1 |
| Indian | 4.1 | 3.4 | 5.2 | 5.2 | 5.4 | 4.7 |
| Pakistani/Bangladeshi | 8.5 | 7.5 | 15.5 | 12.7 | 9.6 | 22.1 |
| Other | 5.0 | 5.1 | 4.3 | 6.7 | 7.1 | 5.4 |
| **Urbanicity** |  |  |  |  |  |  |
| Urban | 79.6 | 79.2 | 82.3 | 85.5 | 85.1 | 86.6 |
| Rural | 20.4 | 20.8 | 17.8 | 14.5 | 14.9 | 13.4 |
| **Parental composition** |  |  |  |  |  |  |
| Two parent household | 70.9 | 71.6 | 65.6 | 60.9 | 60.2 | 63.1 |
| Single parent household | 29.2 | 28.4 | 34.4 | 39.1 | 39.8 | 36.9 |
| **Numb siblings in the Hh** |  |  |  |  |  |  |
| None | 28.7 | 30.7 | 14.9 | 45.4 | 51.9 | 26.1 |
| 1 | 36.7 | 36.3 | 39.2 | 26.2 | 24.7 | 30.8 |
| 2 | 21.0 | 20.6 | 24.4 | 15.7 | 13.5 | 22.2 |
| 3 or more | 14.0 | 12.4 | 21.5 | 12.7 | 9.9 | 20.9 |
| **Household income** |  |  |  |  |  |  |
| Highest | 15.2 | 16.3 | 7.8 | 12.1 | 13.9 | 6.4 |
| Second quintile | 19.8 | 20.7 | 13.5 | 17.0 | 18.8 | 11.4 |
| Third quintile | 20.7 | 21 | 18.5 | 19.1 | 18.7 | 20.1 |
| Fourth quintile | 21.6 | 20.6 | 28.7 | 21.4 | 19.4 | 27.8 |
| Lowest | 22.7 | 21.4 | 31.4 | 30.4 | 29.2 | 34.4 |
| **Parental occupational class** |  |  |  |  |  |  |
| Managerial/professional | 40.9 | 43.1 | 26.4 | 32.8 | 35.7 | 23.8 |
| Intermediate | 19.8 | 20.3 | 16.8 | 19.3 | 20.6 | 15.4 |
| Routine | 23.9 | 23.7 | 25.1 | 26.9 | 28.2 | 23.0 |
| Not in paid work | 15.4 | 13.0 | 31.6 | 21.0 | 15.5 | 37.8 |
| **Parental education** |  |  |  |  |  |  |
| Degree | 31.9 | 33.3 | 22.5 | 27.4 | 29.3 | 21.3 |
| Other Higher | 15.0 | 15.0 | 14.6 | 13.6 | 13.8 | 13.0 |
| A-Level | 22.1 | 22.4 | 20 | 25.2 | 26.0 | 22.7 |
| GSCE | 19.5 | 19.0 | 23.1 | 19.4 | 18.3 | 22.9 |
| Other qualification | 5.5 | 5.1 | 8.6 | 6.1 | 5.6 | 7.6 |
| No qualification | 6.0 | 5.3 | 11.1 | 8.3 | 7.0 | 12.5 |
| Unweighted distributions: Sample after applying inclusion/exclusion criteria, before propensity score matching (PSM).  Individuals were excluded from the analysis due to one of the following reasons: (i) carers missing outcome measures both pre- and post-onset, (ii) incomplete data on other covariables, or (iii) non-carers with fewer than two outcome measures. | | | | | | |

Table – Descriptive comparison of individuals excluded from the analysis and the analytic sample prior to PS for the self esteem sample

|  | **Pre PSM** | | | | | |
| --- | --- | --- | --- | --- | --- | --- |
|  | **Individuals Included in the PSM** | | | **Individuals Excluded from the PSM** | | |
|  | **total** | **Non carers** | **Carers** | **total** | **Non carers** | **Carers** |
| n | **8,052** | **6,638** | **1,414** | **13,790** | **11,080** | **2,710** |
|  | **%** | **%** | **%** | **%** | **%** | **%** |
| **Gender** |  |  |  |  |  |  |
| Male | 48.6 | 48.2 | 50.8 | 49.8 | 49.4 | 51.33 |
| Female | 51.4 | 51.8 | 49.2 | 50.2 | 50.6 | 48.7 |
| **Age first observed** |  |  |  |  |  |  |
| 10 to 17 | 99.2 | 99.1 | 99.8 | 59.0 | 54.2 | 78.7 |
| 18 to 25 | 0.8 | 0.9 | 0.2 | 41.0 | 45.8 | 21.3 |
| **Ethnicity** |  |  |  |  |  |  |
| White | 76.2 | 77.7 | 69.2 | 69.7 | 71.9 | 60.43 |
| Black | 6.8 | 6.6 | 7.9 | 9.8 | 9.7 | 10.0 |
| Indian | 4.0 | 3.8 | 5.0 | 4.1 | 3.9 | 4.7 |
| Pakistani/Bangladeshi | 8.2 | 7.1 | 13.6 | 10.6 | 8.3 | 19.9 |
| Other | 4.8 | 4.9 | 4.2 | 5.9 | 6.1 | 5.1 |
| **Urbanicity** |  |  |  |  |  |  |
| Urban | 76.8 | 76.2 | 79.7 | 82.5 | 81.6 | 85.95 |
| Rural | 23.2 | 23.8 | 20.3 | 17.5 | 18.4 | 14.1 |
| **Parental composition** |  |  |  |  |  |  |
| Two parent household | 74.6 | 76.1 | 68.0 | 63.8 | 64.0 | 62.8 |
| Single parent household | 25.4 | 24.0 | 32.0 | 36.2 | 36.0 | 37.3 |
| **Number of siblings in the Hh** |  |  |  |  |  |  |
| None | 13.6 | 13.8 | 12.8 | 28.8 | 31.2 | 19.3 |
| 1 | 44.1 | 45.0 | 39.6 | 34.9 | 34.9 | 34.9 |
| 2 | 26.2 | 26.2 | 26.5 | 20.9 | 20.2 | 23.4 |
| 3 or more | 16.1 | 15.0 | 21.1 | 15.4 | 13.6 | 22.5 |
| **Household income** |  |  |  |  |  |  |
| Highest | 16.3 | 17.8 | 9.5 | 11.4 | 12.7 | 6.14 |
| Second quintile | 19.5 | 20.6 | 14.1 | 16.6 | 17.9 | 11.4 |
| Third quintile | 21.0 | 21.6 | 18.5 | 19.5 | 19.6 | 19.2 |
| Fourth quintile | 22.4 | 21.2 | 28.1 | 22.7 | 21.4 | 28.0 |
| Lowest | 20.9 | 19.0 | 29.8 | 29.8 | 28.5 | 35.2 |
| **Parental occupational class** |  |  |  |  |  |  |
| Managerial/professional | 44.2 | 47.7 | 27.8 | 34.3 | 36.7 | 24.6 |
| Intermediate | 19.4 | 19.6 | 18.4 | 19.3 | 20.2 | 15.5 |
| Routine | 20.6 | 19.5 | 25.7 | 26.0 | 26.7 | 23.5 |
| Not in paid work | 15.8 | 13.2 | 28.1 | 20.4 | 16.4 | 36.5 |
| **Parental education** |  |  |  |  |  |  |
| Degree | 35.7 | 38.0 | 25.0 | 25.0 | 26.1 | 20.68 |
| Other Higher | 15.6 | 15.7 | 15.4 | 14.5 | 14.8 | 13.2 |
| A-Level | 19.7 | 19.8 | 19.2 | 25.2 | 25.8 | 22.4 |
| GSCE | 19.3 | 18.4 | 23.6 | 20.6 | 20.2 | 22.4 |
| Other qualification | 4.6 | 4.0 | 7.5 | 6.8 | 6.4 | 8.8 |
| No qualification | 5.1 | 4.2 | 9.3 | 7.9 | 6.8 | 12.5 |
| Unweighted distributions: Sample after applying inclusion/exclusion criteria, before propensity score matching (PSM).  Individuals were excluded from the analysis due to one of the following reasons: (i) carers missing outcome measures both pre- and post-onset, (ii) incomplete data on other covariables, or (iii) non-carers with fewer than two outcome measures. | | | | | | |

Table – Descriptive comparison of individuals excluded from the analysis and the analytic sample prior to PS for the self-rated health sample

|  | **Pre PSM** | | | | | |
| --- | --- | --- | --- | --- | --- | --- |
|  | **Individuals Included in the PSM** | | | **Individuals Excluded from the PSM** | | |
|  | **total** | **Non carers** | **Carers** | **total** | **Non carers** | **Carers** |
| n | **19,711** | **17,497** | **2,214** | **13,790** | **11,080** | **2,710** |
|  | **%** | **%** | **%** | **%** | **%** | **%** |
| **Gender** |  |  |  |  |  |  |
| Male | 48.2 | 47.9 | 50.1 | 50.6 | 50.4 | 51.2 |
| Female | 51.8 | 52.1 | 49.9 | 49.4 | 49.6 | 48.9 |
| **Age** |  |  |  |  |  |  |
| 10 to 17 | 61.3 | 58.3 | 85.1 | 50.8 | 41.9 | 73.1 |
| 18 to 25 | 38.7 | 41.7 | 14.9 | 49.2 | 58.1 | 26.9 |
| **Ethnicity** |  |  |  |  |  |  |
| White | 73.9 | 74.9 | 65.5 | 67.4 | 70.4 | 60.0 |
| Black | 7.9 | 7.9 | 8.3 | 9.1 | 8.8 | 9.6 |
| Indian | 4.3 | 4.1 | 5.7 | 4.8 | 5.1 | 4.2 |
| Pakistani/Bangladeshi | 8.8 | 7.8 | 16.2 | 12.3 | 8.8 | 20.9 |
| Other | 5.2 | 5.2 | 4.4 | 6.4 | 6.9 | 5.2 |
| **Urbanicity** |  |  |  |  |  |  |
| Urban | 80.0 | 79.6 | 82.7 | 85.0 | 84.7 | 85.8 |
| Rural | 20.0 | 20.4 | 17.3 | 15.0 | 15.3 | 14.2 |
| **Parental composition** |  |  |  |  |  |  |
| Two parents household | 70.3 | 70.9 | 65.5 | 61.5 | 60.8 | 63.5 |
| Single parent household | 29.7 | 29.1 | 34.6 | 38.5 | 39.3 | 36.5 |
| **Number of siblings in the Hh** |  |  |  |  |  |  |
| None | 30.6 | 32.5 | 15.6 | 42.0 | 49.4 | 24.4 |
| 1 | 35.9 | 35.6 | 38.9 | 27.2 | 25.3 | 31.7 |
| 2 | 20.4 | 19.8 | 24.9 | 16.9 | 14.7 | 21.9 |
| 3 or more | 13.1 | 12.1 | 20.6 | 14.0 | 10.6 | 22.0 |
| **Household income** |  |  |  |  |  |  |
| Highest | 15.4 | 16.3 | 8.4 | 11.5 | 13.7 | 6.0 |
| Second quintile | 20 | 20.9 | 13.4 | 16.2 | 18.0 | 11.7 |
| Third quintile | 20.6 | 20.9 | 18.1 | 19.2 | 18.7 | 20.4 |
| Fourth quintile | 21.1 | 20.1 | 28.4 | 22.7 | 20.6 | 28.1 |
| Lowest | 22.9 | 21.8 | 31.7 | 30.4 | 29.0 | 33.9 |
| **Parental occupational class** |  |  |  |  |  |  |
| Managerial/professional | 40.6 | 45.2 | 26.5 | 32.8 | 36.5 | 24.0 |
| Intermediate | 19.7 | 20.2 | 16.3 | 19.5 | 20.9 | 16.1 |
| Routine | 24.1 | 24.1 | 24 | 26.5 | 27.4 | 24.4 |
| Not in paid work | 15.6 | 13.2 | 33.2 | 21.1 | 15.3 | 35.5 |
| **Parental educational** |  |  |  |  |  |  |
| Degree | 31.6 | 32.7 | 23.2 | 27.7 | 30.4 | 20.7 |
| Other Higher | 14.9 | 15 | 14.3 | 13.7 | 13.8 | 13.4 |
| A-Level | 22 | 22.3 | 19.7 | 25.6 | 26.7 | 22.9 |
| GSCE | 19.6 | 19.1 | 23 | 19.3 | 17.7 | 23.1 |
| Other qualification | 5.6 | 5.2 | 8.5 | 6.0 | 5.3 | 7.8 |
| No qualification | 6.3 | 5.7 | 11.4 | 7.9 | 6.2 | 12.1 |
| Unweighted distributions: Sample after applying inclusion/exclusion criteria, before propensity score matching (PSM).  Individuals were excluded from the analysis due to one of the following reasons: (i) carers missing outcome measures both pre- and post-onset, (ii) incomplete data on other covariables, or (iii) non-carers with fewer than two outcome measures.  Hh: Household | | | | | | |

**Appendix 15 – Sensitivity analysis applying survey weights**

Shown below are the results of sensitivity analysis applying the baseline (wave at first observation) cross-sectional survey weights to the main analyses comparing the health and wellbeing trajectories of carers vs non-carers. The findings with the weights applied are the same as the unweighted analyses presented in the main manuscript.

Figure – Changes in life satisfaction before, during and after becoming a young carer including weights

**
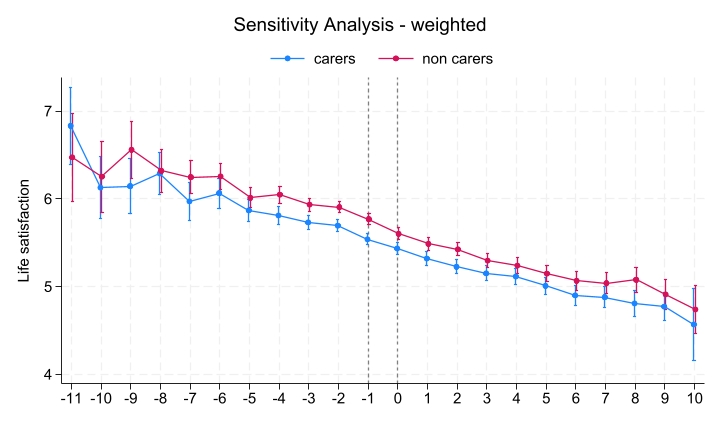
**

Figure – Changes in self esteem before, during and after becoming a young carer including weights

**
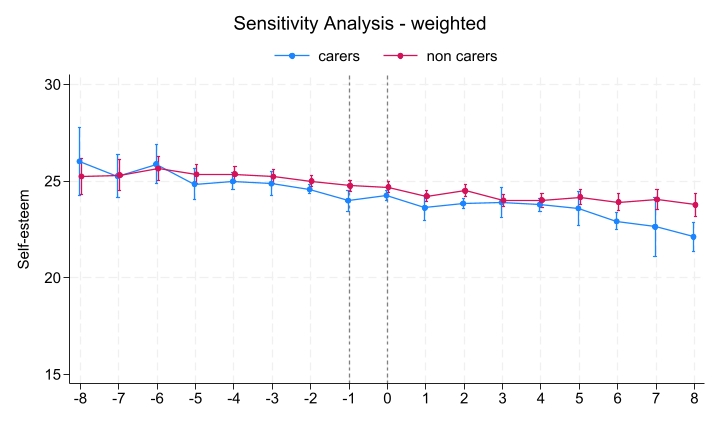
**

Figure – Changes in self-rated before, during and after becoming a young carer including weights

**
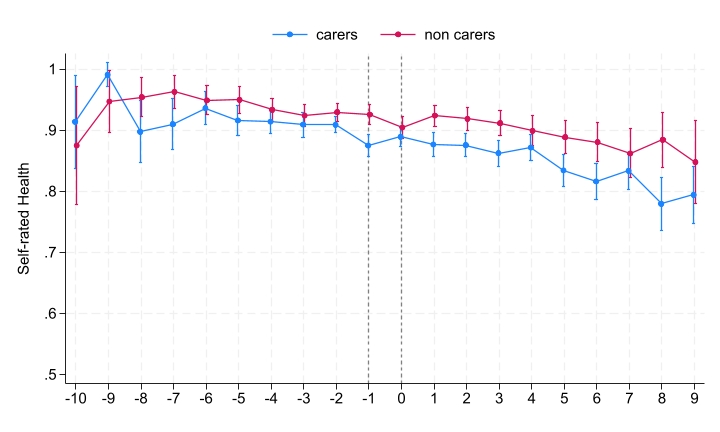
**
